# A Vacuum Vapor Deposition Strategy to Fe Single‐Atom Catalysts with Densely Active Sites for High‐Performance Zn–Air Battery

**DOI:** 10.1002/advs.202306594

**Published:** 2024-05-15

**Authors:** Xiang Yang, Baohui Zhu, Zhiyang Gao, Can Yang, Jingbo Zhou, Aijuan Han, Junfeng Liu

**Affiliations:** ^1^ State Key Laboratory of Chemical Resource Engineering Beijing University of Chemical Technology Beijing 100029 P. R. China; ^2^ Baidu Research Haidian District Beijing 100193 P. R. China

**Keywords:** 2D material, active site engineering, oxygen reduction reaction, single‐atom catalyst, Zn–air battery

## Abstract

Iron single‐atom catalysts (SACs) have garnered increasing attention as highly efficient catalysts for the oxygen reduction reaction (ORR), yet their performance in practical devices remains suboptimal due to the low density of accessible active sites. Anchoring iron single atoms on 2D support is a promising way to increase the accessible active sites but remains difficult attributing to the high aggregation tendency of iron atoms on the 2D support. Herein, a vacuum vapor deposition strategy is presented to fabricate an iron SAC supported on ultrathin N‐doped carbon nanosheets with densely active sites (FeSAs‐UNCNS). Experimental analyses confirm that the FeSAs‐UNCNS achieves densely accessible active sites (1.11 × 10^20^ sites g^−1^) in the configuration of Fe─N_4_O. Consequently, the half‐wave potential of FeSAs‐UNCNS in 0.1 m KOH reaches a remarkable value of 0.951 V versus RHE. Moreover, when employed as the cathode of various kinds of Zn–air batteries, FeSAs‐UNCNS exhibits boosting performances by achieving a maximum power density of 306 mW cm^−2^ and long cycle life (>180 h) at room temperature, surpassing both Pt/C and reported SACs. Further investigations reveal that FeSAs‐UNCNS facilitates the mass and charge transfer during catalysis and the atomic configuration favors the desorption of *OH kinetically.

## Graphical abstract

An iron single‐atom catalyst supported on ultrathin N‐doped carbon nanosheets with densely active sites is prepared by vacuum vapor deposition. Due to the high density of accessible active sites and atomically dispersed Fe─N_4_O configuration, this iron catalyst exhibits remarkable performance in alkaline oxygen reduction reactions and various zinc–air batteries including primary, secondary, and seawater‐based ones.

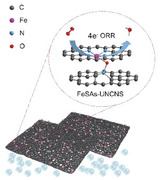

## 1 Introduction

Zn–air batteries find significant applications in various devices including wearable devices, communication base stations, and electric vehicles.^[^ ^1^ ^]^ The primary challenge of these batteries lies in the sluggish kinetics of the cathode oxygen reduction reaction (ORR).^[^ ^2^ ^]^ While Pt/C catalysts are currently used commercially as ORR catalysts, they suffer from high cost and poor stability.^[^ ^3^ ^]^ Therefore, many research efforts have been put into developing efficient non‐precious metal catalysts to substitute Pt/C.^[^ ^4^ ^]^ Among them, iron single‐atom catalysts (SACs) supported on nitrogen‐doped carbon (NC), i.e., Fe─NC catalysts, have emerged as the most promising alternative.^[^ ^5^ ^]^ However, their performance in practical devices is still unsatisfactory. Currently, Fe─NC catalysts are obtained via a high‐temperature pyrolysis procedure that usually renders limited control over the final distribution of FeN_x_ moieties in the NC matrix.^[^ ^6^ ^]^ The micropore‐hosted Fe─N_x_ moieties near the external surface of the catalysts participate better in the ORR, while those buried deep within a dense matrix remain inactive.^[^ ^7^ ^]^ Therefore, the ORR activity of Fe─NC catalysts is predominantly correlated with their site density (SD, the number of accessible active sites per gram of catalyst) which is proportional to the Fe loading and the utilization rate (the proportion of accessible FeN_x_ over the total FeN_x_).^[^ ^7^, ^8^ ^]^ When the Fe loading increased, the utilization rate of their Fe─NC catalyst dropped, as revealed by Shui et al.^[^ ^7^ ^]^ They achieved a maximum SD of 3.4 × 10^19^ sites g^−1^, which was only 0.3 wt.% in the total catalyst.^[^ ^7^ ^]^ There is still a big space to increase the SD of Fe─NC, but remains a big challenge.

2D nanomaterials have attracted extensive attention in the field of catalysis due to their 1D thinness, which facilitates enhanced diffusion and mass transfer during catalytic processes.^[^ ^9^ ^]^ In particular, ultrathin 2D materials with a thickness of only single or a few atoms (typically >5 nm) exhibit unique properties and activities attributed to their large specific surface area, and high ratio of surface atoms to entire atoms.^[^ ^9^ ^]^ For instance, our group prepared a Zn single‐atom catalyst on 1 nm‐thick nanosheet and found it achieving a remarkably high half‐wave potential of 0.91 V versus RHE in alkaline ORR, much higher than its bulky 2D counterpart.^[^ ^10^ ^]^ Anchoring single atoms on ultrathin 2D supports can increase the micropore‐hosted Fe─N_x_ moieties near the external surface, and is a promising way to optimize ORR performance. However, due to the high tendency of metal atoms to aggregate into nanoparticles or nanoclusters, synthesizing ultrathin 2D NC nanosheet‐supported Fe single‐atom catalysts remain rather difficult, with only a few works reported but having limited Fe loading (<1.5 wt.%).^[^ ^11^ ^]^ Therefore, it is highly desirable yet challenging to construct ultrathin 2D NC‐supported Fe single‐atom catalysts with high metal loading to achieve highly accessible active sites. Recently, zeolitic imidazole framework (ZIF‐8), as an exemplary precursor possessing enriched nitrogen and a unique porous structure, shines as the precursor for Fe single‐atom catalysts with high loadings.^[^ ^7^, ^12^ ^]^ Inspired by these studies and considering that ZIF‐L shares similar component and structure features with ZIF‐8 but possesses distinct 2D morphology, we propose utilizing ultrathin ZIF‐L nanosheet as an ideal precursor to address the issue of insufficient accessible active sites in Fe─NC.

Herein, we developed a Fe single‐atom catalyst supported on ultrathin nitrogen‐doped carbon nanosheets (FeSAs‐UNCNS) with densely accessible active sites by a vacuum vapor deposition (VVD) strategy using ultrathin ZIF‐L (U‐ZIF‐L) nanosheets as the precursor. Iron acetylacetonate molecules were drilled in the micropores of U‐ZIF‐L by the VVD process, and transformed into FeSAs‐UNCNS after pyrolysis. Aberration‐corrected high‐angle annular dark‐field scanning transmission electron microscopy (AC HAADF‐STEM) and X‐ray absorption fine structure (XAFS) results confirmed the atomic dispersion of iron atoms in the form of Fe─N_4_O. Benefiting from the ultrathin nature of the NC, the SD of FeSAS‐UNCNS achieved as high as 1.11 × 10^20^ site g^−1^, with a Fe loading amount of 4.22 wt.%. Impressively, this FeSAs‐UNCNS demonstrated a boosting ORR performance with a half‐wave potential of 0.951 V versus RHE in alkaline media. Furthermore, the Zn–air batteries assembled using this FeSAs‐UNCNS showed excellent performance for primary, seawater‐based, and rechargeable Zn–air batteries, with a record‐high maximum peak power density (306 mW cm^−2^) and long cycle life (>180 h) at room temperature. This work not only provides an efficient method for synthesizing 2D single‐atom catalysts with densely accessible active sites but also sheds light on achieving high‐performance Zn–air batteries through active site engineering.

## 2 Results and Discussion

### 2.1 Morphological Characterization of FeSAs‐UNCNS

**Figure 1:**
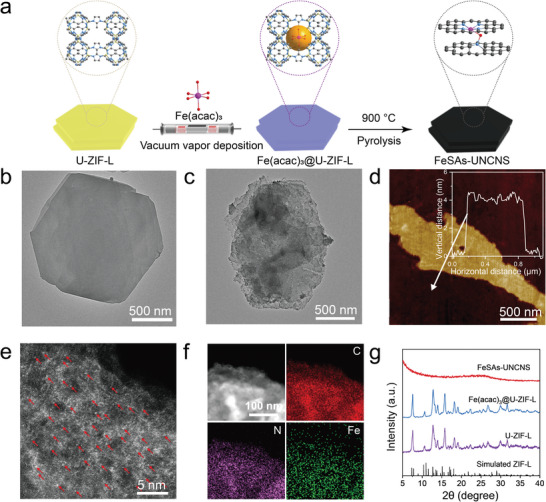
Synthesis and characterizations of FeSAs‐UNCNS. a) Synthetic scheme for FeSAs‐UNCNS. The TEM image of b) U‐ZIF‐L and c) FeSAs‐UNCNS. d) The AFM image of FeSAs‐UNCNS. e) The AC‐HAADF‐STEM image of FeSAs‐UNCNS (numerous Fe atoms are indicated by red arrows to facilitate identification). f) EDX mapping of FeSAs‐UNCNS, C (red), N (purple), and Fe (green). g) XRD patterns of simulated ZIF‐L, U‐ZIF‐L, Fe(acac)_3_@U‐ZIF‐L and FeSAs‐UNCNS.

The FeSAs‐UNCNS catalyst was prepared by a VVD strategy (**Figure** **1**a). Typically, U‐ZIF‐L was prepared by mixing zinc nitrate aqueous solution with 2‐methylimidazole aqueous solution, while 1‐methylimidazole was added to help the formation of ultrathin nanosheets.^[^ ^10^ ^]^ Second, the U‐ZIF‐L was put in a sealed tube (the setup was schematically illustrated in Figure 1a) for VVD of iron acetylacetonate (i.e., Fe(acac)_3_) at 200 °C to form Fe(acac)_3_@U‐ZIF‐L. In this step, the Fe(acac)_3_ was chosen due to its low boiling point (187.6 °C), stable nature at high temperature, and adequate molecular size (9.7 Å; Figure S1, Supporting Information). It was sublimated into vapor and drilled into the microporous cavity of U‐ZIF‐L (with cavity size of 11.9 Å). Due to the size effect, one cavity can only accommodate one Fe(acac)_3_ molecule.^[^ ^13^ ^]^ Nitrogen adsorption–desorption isotherm showed that the surface area of U‐ZIF‐L decreased from 123.8 to 59.0 m^2^ g^−1^ after this VVD step, and the micropores largely decreased while the volumes of mesopores and macropores remained unchanged (Table S1 and Figure S2, Supporting Information). It testified that Fe(acac)_3_ molecules successfully drilled into the micropores. Finally, FeSAs‐UNCNS was obtained by topo‐conversion of Fe(acac)_3_@U‐ZIF‐L at 900 °C for 3 h under Ar atmosphere. Electron microscopes were taken to characterize the morphology of samples. The U‐ZIF‐L had a typical 2D nanosheet morphology with diameters of 1–3 µm (Figure 1b; Figure S3, Supporting Information), and the thickness was ≈4.0 nm as measured by atomic force microscope (AFM) (Figure S4, Supporting Information). After VVD process, the Fe(acac)_3_@U‐ZIF‐L retained the 2D nanosheet morphology (Figure S5, Supporting Information). The final product FeSAs‐UNCNS preserved the ultrathin nanosheet morphology (Figure 1c,d). No nanoparticles or clusters were detected in the high‐resolution transmission electron microscopy (HRTEM) images (Figure S6, Supporting Information). Only discrete and dense bright spots were identified on the AC HAADF‐STEM image (Figure 1e). Moreover, the energy dispersive X‐ray spectroscopy (EDX) elemental mapping of the FeSAs‐UNCNS indicated that Fe, C, and N elements were uniformly distributed throughout the support (Figure 1f). The X‐ray diffraction (XRD) pattern and Raman spectra testified the presence of graphitic carbon (Figure 1g; Figure S7, Supporting Information).^[^ ^4^, ^14^ ^]^The Fe loading was determined to be 4.22 wt.% (Table S2, Supporting Information) by inductively coupled plasma optical emission spectrometry (ICP‐OES), which is among the highest level in Fe SACs.^[^ ^12^, ^15^ ^]^ The BET surface area of FeSAs‐UNCNS was 1018 m^2^ g^−1^ (Figure S8 and Table S1, Supporting Information).

### 2.2 Fine Structure of FeSAs‐UNCNS

**Figure 2:**
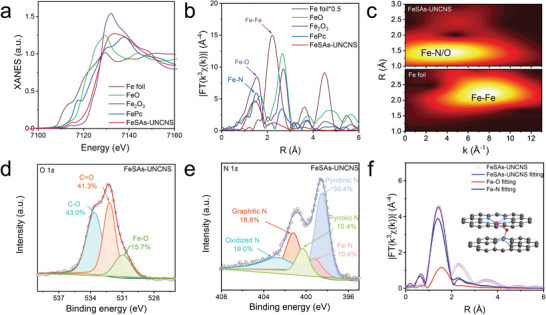
XAFS and XPS characterization of FeSAs‐UNCNS. a) XANES spectra and b) Fourier transform at the Fe K‐edge of FeSAs‐UNCNS, FePc, Fe_2_O_3_, and Fe foil. c) WT of FeSAs‐UNCNS in comparison with Fe foil samples. High‐resolution d) O 1s and e) N 1s XPS spectra for FeSAs‐UNCNS. f) The EXAFS fitting curve at *R* space and schematic model of FeSAs‐UNCNS (Fe: purple, N: blue, O: red, and C: gray).

X‐ray absorption spectroscopy (XAS) was performed to characterize the detailed atomic structure of FeSAs‐UNCNS.^[^ ^12^ ^]^ The X‐ray absorption near edge structure (XANES) spectra of FeSAs‐UNCNS at Fe K‐edge were given in **Figure** **2**a, with Fe foil, iron phthalocyanine (FePc), FeO, and Fe_2_O_3_ as references. The absorption edge of FeSAs‐UNCNS was close to that of Fe_2_O_3_, indicating that the Fe atom is positively charged and the valence state is close to +3. The average valence state of Fe in the FeSAs‐UNCNS was ≈+2.85, as revealed from the fitted average oxidation states of Fe foil, FePc, and Fe_2_O_3_ obtained from the analysis of Fe K‐edge XANES (Figure S9, Supporting Information). It agreed well with the high‐resolution Fe 2p X‐ray photoelectron spectroscopy (XPS) spectra of FeSAs‐UNCNS (Figure S10, Supporting Information). In the Fourier transform extended X‐ray absorption fine structure (FT‐EXAFS), FeSAs‐UNCNS exhibited a prominent peak at ≈1.5 Å (Figure 2b, without phase correction) which could be attributed to Fe─N/O scattering paths.^[^ ^16^ ^]^ No obvious peaks assigned to the Fe─Fe bond were detected in FeSAs‐UNCNS. In addition, wavelet transform (WT) were further conducted.^[^ ^17^ ^]^ No intensity maximum at 2.7 Å^−1^ indexed to Fe─Fe coordination was observed in the WT of FeSAs‐UNCNS as compared to the WT of Fe foil, while the only intensity maximum at ≈3.8 Å^−1^ that may be ascribed to Fe─N/O contribution (Figure 2c). Furthermore, quantitative structural configuration was analyzed through a least‐squares EXAFS fitting. Considering the coexistence of Fe─N and Fe─O peaks in the XPS spectra and Fe─O bond length in optimized configurations (Figure 2d,e; Figures S11–S13, Supporting Information),^[^ ^12^ ^]^ the best fitting model was proposed with one Fe atom coordinated by four N atoms and one axial O atom bonded to the second graphene layer (Figure 2f inset, Table S3, Supporting Information).

### 2.3 Quantitative Characterization of the Accessible Active Sites

**Figure 3:**
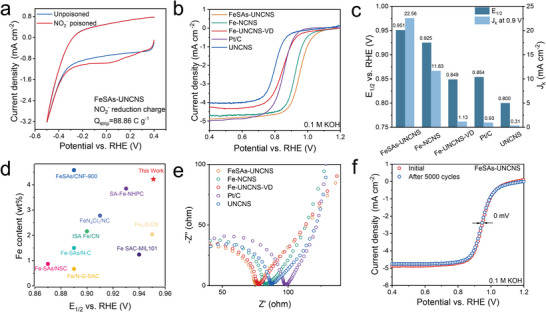
In situ SD evaluation and ORR performance of the samples. a) Nitrite stripping voltammetry of FeSAs‐UNCNS in N_2_‐saturated 0.5 m acetate electrolyte buffer (pH 5.2). b) LSV curves recorded in an O_2_‐saturated 0.1 m KOH solution at a rotation speed of 1600 rpm and a sweep rate of 10 mV s^−1^. c) Comparison of *J*_k_ and *E*_1/2_ for different catalysts. d) Fe content measured by ICP‐OES and *E*_1/2_ compared with literature. e) Nyquist plots of EIS in 0.1 m KOH. f) ORR polarization curves of FeSAs‐UNCNS before and after 5000 cycles.

Site density was quantitatively measured by an in situ electrochemical method using nitrite as the probe.^[^ ^18^ ^]^ NO group was strongly bonded to Fe sites in nitrite solution and transformed into NH_3_ upon electroreduction with five electrons transfer per NO group, therefore the SD can be estimated from the electric charge needed during this reduction process.^[^ ^18^ ^]^ No reduction peak was observed for the sample UNCNS that was prepared by a similar procedure of FeSAs‐UNCNS but without Fe(acac)_3_ (Figure S14, Supporting Information), excluding the interference of nitrogen‐doped carbon support in the calculation of SD. FeSAs‐UNCNS achieved an SD of 1.11 × 10^20^ sites g^−1^ (**Figure** **3**a; Table S2, Supporting Information), which is among the highest level for Fe single‐atom catalysts in literatures and beats all 2D supported ones (Table S4, Supporting Information). Next, the conditions of FeSAs‐UNCNS to host the accessible active sites was investigated. Two catalysts were prepared by a similar method to FeSAs‐UNCNS except using much thicker ZIF‐L nanosheets (the obtained catalyst was denoted as Fe─NCNS) and without vacuum in the VVD process (the obtained catalyst was denoted as Fe‐UNCNS‐VD), respectively. In the former case, 200‐nm‐thick ZIF‐L nanosheets were used instead of the U‐ZIF‐L (Figure S15, Supporting Information), resulting in the formation of Fe nanoparticles on thick nanosheets due to their tendency to aggregate even though the Fe loading was smaller than that of FeSAs‐UNCNS (Figure S16, Table S2, Supporting Information).^[^ ^19^ ^]^ The SD of Fe─NCNS (5.02 × 10^19^ sites g^−1^), highlights the advantage of ultrathin nanosheet support in anchoring Fe atoms. For the latter, the Fe loading was only 0.09 wt.% without the vacuum during VVD process, as tiny Fe(acac)_3_ molecules were drilled in the micropores deduced from the much lighter color change (Figure S17, Table S2, Supporting Information). FeSAs‐UNCNS‐VD only gained an SD of only 0.88 × 10^19^ sites g^−1^, demonstrating the necessity of high loading through vacuum treatment. This finding confirmed that both ultrathin structure and high Fe loading enabled by the VVD strategy contribute to the densely accessible active sites.

### 2.4 ORR Performance

The ORR activity of FeSAs‐UNCNS was then evaluated in a three‐electrode system at room temperature, together with commercial Pt/C catalyst and the controlled Fe electrocatalysts for comparison. In the linear sweep voltammetry (LSV) curve (Figure 3b), the FeSAs‐UNCNS delivered extremely impressive ORR onset potential (*E*_onset_) of 1.10 V versus RHE and half‐wave potential (*E*_1/2_) of 0.951 V versus RHE, while the UNCNS prepared by pyrolysis of U‐ZIF‐L nanosheets directly only showed an *E*_1/2_ of 0.800 V versus RHE, indicating that the excellent activity of FeSAs‐UNCNS was mainly attributed to the atomically dispersed Fe sites. The *E*_1/2_ and kinetic current density at 0.90 V (*J*_k_ at the rate of 0.90 V) followed the same trend of SD for the Fe‐based electrocatalysts. *E*_1/2_ and kinetic current density at 0.90 V (*J*_k_ at the rate of 0.90 V) of FeSAs‐UNCNS (*E*_1/2_ = 0.951 V, and *J*_k_ at the rate of 0.90 V = 22.6 mA cm^−2^) were significantly superior to those of Pt/C (*E*_1/2_ = 0854 V, and *J*_k_ at the rate of 0.90 V = 0.93 mA cm^−2^), Fe─NCNS (*E*_1/2_ = 0.925 V, and *J*_k_ at the rate of 0.90 V = 11.6 mA cm^−2^), Fe‐UNCNS‐VD (*E*_1/2_ = 0.850 V, and *J*_k_ at the rate of 0.90 V = 1.1 mA cm^−2^) (Figure 3c). A typical Fe single‐atom catalyst derived from ZIF‐8 and Fe(acac)_3_ precursors (FeSAs‐NC) was synthesized following literature procedures.^[^ ^8^ ^]^ The FeSAs‐NC demonstrated an *E*_1/2_ of 0.907 V versus RHE and a *J*_k_ at the rate of 0.90 V of 6.37 mA cm^−2^ (Figure S18, Supporting Information), smaller than those of FeSAs‐UNCNS. The SD of FeSAs‐NC was determined to be 8.03 × 10^19^ sites g^−1^ (Table S2, Supporting Information), which was smaller than that of FeSAs‐UNCNS. It suggested that the ultrathin 2D support promotes the exposure of active sites, resulting in improved ORR activity. Impressively, the *E*_1/2_ of FeSAs‐UNCNS was among the highest level in literature (Figure 3d).^[^ ^12^, ^15^ ^]^ The electrochemical surface area (ECSA) and electrochemical impedance spectroscopy (EIS) were then investigated for the Fe‐electrocatalysts. FeSAs‐UNCNS showed a larger ECSA of 1417 cm^2^, which may benefit to the densely exposed active sites and mass transfer during catalyst (Figures S19,S20, and Table S5, Supporting Information).^[^ ^20^ ^]^ Furthermore, in order to eliminate the influence of ECSA on the ORR performance, the *J*_k_ values of FeSAs‐UNCNS, FeNPs‐UNCNS, and FeSAs‐UNCNS‐VD were normalized by their corresponding measured ECSA (Figure S21, Supporting Information).^[^ ^21^ ^]^ The normalized *J*_k_ curves based on ECSA exhibited a consistent trend with the *J*_k_ in Figure 3c, further confirming the exceptional intrinsic activity of FeAs‐UNCNS toward ORR. The smaller semicircle diameter in the EIS spectra of FeSAs‐UNCNS indicated a lower charge transfer resistance for FeSAs‐UNCNS, which may favor the charge transfer process during ORR.^[^ ^10^ ^]^ The iron loading was controllable through tuning the amount of Fe(acac)_3_ in the VVD process. With the increasing amount of Fe(acac)_3_ used, the iron loading increased (Table S6, Supporting Information). The best ORR performance was obtained for FeSAs‐UNCNS with 100 mg of Fe(acac)_3_ used. A higher amount of Fe(acac)_3_ resulted in the aggregation of iron atoms. FeNPs‐UNCNS prepared using 140 mg of Fe(acac)_3_ had obvious Fe nanoparticles on the UNCNS support (Figures S22 and S23, Supporting Information), and showed a lower *E*_1/2_ and *J*_k_@0.90 V (Figure S24, Supporting Information). The similar semicircle diameter of FeNPs‐UNCNS and FeSAs‐UNCNS in EIS spectra indicated the advantage of the atomically dispersed Fe active sites. Other iron sources such as iron nitrate and iron sulfate were also used as the iron precursors. These inorganic salts are not stable at high temperatures and they decompose at temperatures higher than their boiling points. Therefore, these inorganic salts negligibly transferred to the U‐ZIF‐L during the vacuum vapor deposition step. The best ORR performance was achieved by FeSAs‐UNCNS using iron (III) acetylacetonate (Figure S25, Supporting Information). Moreover, the electron transfer number *n* obtained from the Koutecky–Levich (K–L) plots were 3.87–4.00 while only <2.0% hydrogen peroxide was observed in the rotating ring‐disk electrode (RRDE), suggesting the high selectivity of FeSAs‐UNCNS for 4e^−^ reduction (Figure S26, Supporting Information).^[^ ^12^ ^]^ FeSAs‐UNCNS exhibited outstanding tolerance to methanol crossover (Figure S27, Supporting Information) and excellent stability (Figure 3f). After 5000 cycles, no obvious decay in *E*_1/2_ and limiting current density was detected, and no iron particles were found in the TEM image after cycling (Figure S28, Supporting Information). All the above analyses indicated that FeSAs‐UNCNS had an outstanding ORR ability, resulting from the higher SD and easier charge and mass transfer.

### 2.5 Computational Calculations

**Figure 4:**
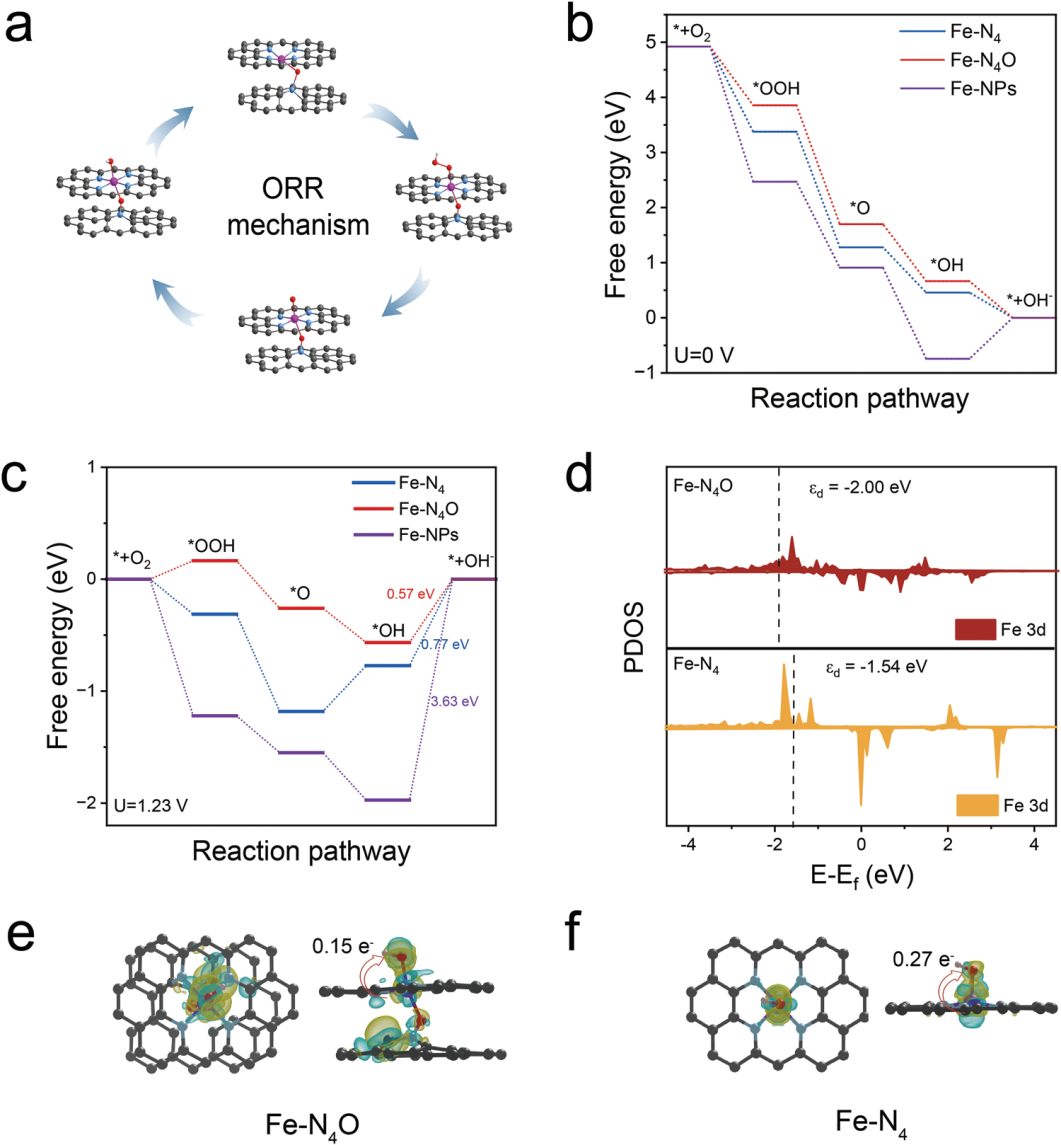
DFT calculations. a) Proposed ORR mechanism on the Fe─N_4_O catalyst. Free energy diagram of ORR at b) *U* = 0 V and c) *U* = 1.23 V of Fe─N_4_, Fe─N_4_O, and Fe─NPs models. d) PDOS plots of the Fe 3d for the Fe─N_4_O and Fe─N_4_ structures. The Fe d‐band center position is indicated by the vertical dashed line. The charge density differences of e) Fe─N_4_O and f) Fe─N_4_ with *OH adsorption. The charge accumulation and depletion are colored in cyan and yellow.

DFT calculations were further carried out to unveil the theoretical mechanism of the outstanding ORR performance of the FeSAs‐UNCNS from the aspect of the active sites. The models of Fe─N_4_O and Fe cluster with six Fe atoms were used to represent the two active sites of atomically Fe active site and Fe nanoparticles in our Fe‐based electrocatalyst, respectively. Meanwhile, a model with typical Fe─N_4_ active site for Fe SACs in literature was also constructed for comparison (Figures S29 and S30, Supporting Information).^[^ ^12^ ^]^ Gibbs free energy distributions were calculated for the four basic steps of the ORR on these three models at 0 and 1.23 V.^[^ ^21^ ^]^ The ORR process involves four reduction steps that accept one electron, respectively (**Figure** **4**a). At *U* = 0 V, all electron transfer steps on Fe─N_4_O and Fe─N_4_ were exothermic (Figure 4b). The final reduction step on Fe─NPs was endothermic, indicating that an external force (that is, the applied voltage) was required to drive this process. As shown in Figure 4c, the potential limiting steps (PLS) of Fe─N_4_O, Fe─N_4_, and Fe─NPs were all the final *OH desorption steps. Among them, Fe─N_4_O had the lowest ∆*G*_PLS_ (the optimal free energy changes of the PLS) values of 0.57 eV (Table S7, Supporting Information), indicating that the electron transfer from the catalyst to the adsorbed *OH species to form OH^−^ was much easier over Fe─N_4_O. In addition, to bridge the relationship between the adsorption interaction and charge transfer of *OH, we calculated the Bader charge (Figure 4e; Table S8, Supporting Information). Fe─N_4_ possessed larger electronic accumulation on *OH and depletion on metal sites.^[^ ^22^ ^]^ Correspondingly, the *OH adsorption on Fe─N_4_ was stronger than that on Fe─N_4_O, which is not easy to desorb. Therefore, Fe─N_4_O possessed significantly high intrinsic activity. To gain into the role of the axial O atoms in the Fe─N_4_O structure in regulating the Fe level and improving the catalytic activity of ORR, the projected density of states (PDOS) were further investigated. The axial bridged O atom made the d‐band center of the Fe 3d orbital in the Fe─N_4_O structure more negative than the d‐band of Fe─N_4_ (Figure 4d). Thus, it made antibonding orbitals more difficult to fill and bond formation less attenuated, which weakened the adsorption of ORR intermediates. Compared with Fe─N_4_, the overlapping peak near −5–−2 eV was significantly reduced (Figure S31, Supporting Information).^[^ ^22^ ^]^ It indicated that the Fe─N_4_O structure weakened the interaction between the metal center and *OH and therefore enhanced the overall dynamics of ORR.

### 2.6 Zn–Air Battery Performance

**Figure 5:**
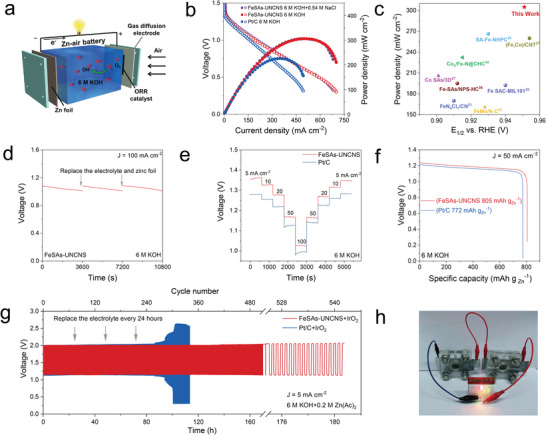
Zn–air battery performance. a) Schematic illustration of an as‐assembled primary Zn–air battery. b) Polarization and power density curves of primary and simulated seawater‐based Zn–air batteries. c) Comparison of maximum power density of Zn–air batteries and *E*_1/2_. d) Long‐time galvanostatic discharge curves at 100 mA cm^−2^. e) Galvanostatic discharge tests at different current densities. f) Voltage changes of replenishing the Zn foil anode and the electrolyte at 100 mA cm^−2^. g) Galvanostatic charge–discharge cycling curves for the rechargeable Zn–air batteries at the current density of 5 mA cm^−2^. (h) Photo of two Zn–air batteries in a series of lighting diodes.

To further explore the practical application, the FeSAs‐UNCNS was employed as the cathode for various Zn–air batteries with commercial Pt/C as a comparison at room temperature (**Figure** **5**). The FeSAs‐UNCNS‐based primary Zn–air battery exhibited a higher stable open‐circuit voltage of 1.483 V than that of the Pt/C‐based battery (1.429 V) (Figure S32, Supporting Information). The discharge voltages for the FeSAs‐UNCNS‐based primary Zn–air battery were also higher than that for Pt/C‐based Zn–air battery at current densities from 5 to 100 mA cm^−2^. The maximum power density for the FeSAs‐UNCNS‐based primary Zn–air battery reached 305.7 mW cm^−2^ at 0.615 V, far exceeding that of Pt/C‐based (226.1 mW cm^−2^ at 0.661 V), all reported iron SACs‐based, and most non‐precious metal‐based Zn–air batteries (Figure 5b,c; Table S9, Supporting Information).^[^ ^15^, ^23^ ^]^ Meanwhile, the specific capacities of Zn–air batteries assembled by FeSAs‐UNCNS at 50 mA cm^−2^ (805 mAh g_Zn_^−1^) were higher than that of Pt/C‐based Zn–air battery (772 mAh g_Zn_^−1^) (Figure 5d).^[^ ^15b^ ^]^ Notably, discharge tests showed that the discharge voltage can be restored when changing the current density to the initial ones (Figure 5e), indicating that FeSAs‐UNCNS‐based Zn–air batteries have excellent rate performance. At a high current density of 100 mA cm^−2^, the voltage can be restored by supplementing the Zn anode and electrolyte every 3600 s, and the stability curve showed superior durability of the FeSAs‐UNCNS (Figure 5f). Additionally, no remarkable voltage drop was observed after 50 000 and 13 000 s when the current discharge experiments were performed at 10 and 50 mA cm^−2^, respectively (Figure S24, Supporting Information), testifying the high stability of the FeSAs‐UNCNS cathode. Using seawater as the water source, seawater Zn–air battery can further reduce the competition with fresh water resources and broaden the application field of Zn–air battery,^[^ ^24^ ^]^ therefore, the seawater Zinc–air battery was also assembled by adding 0.54 m NaCl to simulate seawater composition. The maximum power density of seawater Zn–air battery using FeSAs‐UNCNS as the cathode remained unchanged (Figure 5b), demonstrating its promising potential application on the sea. Moreover, rechargeable Zn–air batteries were also assembled to demonstrate the practical application of our FeSAs‐UNCNS. The rechargeable Zn–air battery for FeSAs‐UNCNS maintained a stable and small (<0.86 V) charge–discharge voltage gap (Δ*V*) over 180 h at 5 mA cm^−2^ (Figure 5g), while the reference rechargeable Zn–air battery for Pt/C showed inferior cycling stability. In addition, the round‐trip efficiency and stability of the rechargeable Zn–air battery for FeSAs‐UNCNS were compatible with that in literature (Table S10, Supporting Information). Besides, connecting two Zn–air batteries in series can easily turn on the diode (Figure 5h), demonstrating the practical application for powering electronics. These results clearly demonstrated that FeSAs‐UNCNS is a promising efficient and stable cathode catalyst to substitute Pt/C in various zinc–air batteries.

## 3 Conclusion

In summary, we have developed an ultrathin nitrogen‐doped carbon‐supported iron single‐atom catalyst with a high density of active sites through a vacuum vapor deposition strategy. The catalyst exhibited a substantial density of accessible active site 1.11 × 10^20^ sites g^−1^, which was in the configuration of Fe─N_4_O. Moreover, the catalyst demonstrated exceptional half‐wave potential and stability in alkaline ORR. Utilizing FeSAs‐UNCNS as the catalyst in Zn–air batteries resulted in remarkable performance surpassing commercial Pt/C, including maximum power density of 306 mW cm^−2^, outstanding cycle life (>180 h), and excellent performance even when operated with seawater electrolyte. This work presents an innovative approach for designing and synthesizing iron atom catalysts with dense active sites to achieving high‐performance Zn–air batteries.

## Conflict of Interest

The authors declare no conflict of interest.

## Author Contributions

X.Y. and B.H.Z. contributed equally to this work. J.F.L. and A.J.H. conceived the project. C.Y. and X.Y. synthesized the materials. B.H.Z., X.Y., and Z.Y.G. conducted the electrocatalytic tests and analyzed the data. X.Y. and J.B.Z. did the DFT calculations. X.Y., B.H.Z., A.J.H., and J.F.L. wrote the manuscript. All authors discussed the results and commented on the manuscript.

## Supporting information

Supporting Information

## Data Availability

The data that support the findings of this study are available from the corresponding author upon reasonable request.
