# Supplementary material for: A Vacuum Vapor Deposition Strategy to Fe Single‐Atom Catalysts with Densely Active Sites for High‐Performance Zn–Air Battery
Source: Adv Sci (Weinh). 2024 May 15;11(34):2306594. doi: 10.1002/advs.202306594 (PMC11425844; doi:10.1002/advs.202306594)
Supplement: Supplementary file 1 — Supporting Information [file ADVS-11-2306594-s001.pdf]

ADVANCED SCIENCE
Open Access

Supporting Information

for *Adv. Sci.*, DOI 10.1002/advs.202306594

A Vacuum Vapor Deposition Strategy to Fe Single-Atom Catalysts with Densely Active Sites for High-Performance Zn–Air Battery

Xiang Yang, Baohui Zhu, Zhiyang Gao, Can Yang, Jingbo Zhou, Aijuan Han and Junfeng Liu**

Supporting Information

A vacuum vapor deposition strategy to Fe single-atom catalysts with densely active sites for high-performance Zn-air battery

Xiang Yang,[+] Baohui Zhu,[+] Zhiyang Gao, Can Yang, Jingbo Zhou, Aijuan Han,* and Junfeng Liu*

Xiang Yang, Baohui Zhu, Zhiyang Gao, Can Yang, Aijuan Han, Junfeng Liu
State Key Laboratory of Chemical Resource Engineering, Beijing University of Chemical Technology, Beijing 100029, PR China
E-mail: ljf@mail.buct.edu.cn; hanaijuan@mail.buct.edu.cn
Jingbo Zhou
Baidu Research, Beijing 100193, PR China

[+] These authors contributed equally to this work.

Table of Contents

Experimental Procedures 3
Chemicals and materials 3
Synthesis procedures 3
Figures and Tables 7
References 31

Experimental Procedures

Chemicals and materials
Zinc nitrate hexahydrate (98%), Nafion (5 wt%), and commercial Pt/C (20 wt%) were purchased from Alfa Aesar. IrO_2 (Macklin), potassium hydroxide (99.99%), 1-methylimidazole (99%), 2-methylimidazole (98%), iron(III) acetylacetonate (98%,), and iron(II) acetylacetonate (98%) were purchased from Aladdin. Sodium nitrite was purchased from Sigma-Aldrich. All chemicals are used without any further purification.

Synthesis procedures
Synthesis of U-ZIF-L
$Zn(NO_3)_2{\cdot}6H_2O$ (360 mg, 1.21 mmol) was dissolved in 80 ml deionized water to form solution A. 2-Methylimidazole (1100 mg, 13.40 mmol) and 1-methylimidazole (1100 mg, 13.40 mmol) were dissolved in 20 ml deionized water to form solution B. Solution A was quickly poured into solution B, and stirred at 25 ^oC for 6 h. The product was washed with ethanol, and dried in an oven at 60 ^oC.
Synthesis of $Fe(acac)_3$@U-ZIF-L
200 mg U-ZIF-L was placed in the porcelain boat in a tube furnace, while 50 mg of $Fe(acac)_3$ was put on both sides. The tube was evacuated to vacuum by a pump. Then furnace was heated to 200 ^oC (5 ^oC min^{-1}), kept for 30 min, and then cooled to room temperature naturally.
Synthesis of FeSAs-UNCNS.
The $Fe(acac)_3$@U-ZIF-L was heated to 900 ^oC (5 ^oC min^{-1}) in an argon atmosphere, and kept for 3 h.
Synthesis of FeSAs-UNCNS-X.
The FeSAs-UNCNS-X were prepared by the similar method to FeSAs-UNCNS except using different amounts of $Fe(acac)_3$ during the vacuum vapor deposition step. X was the total amount of $Fe(acac)_3$ used in the vacuum vapor deposition step.
Synthesis of Fe-UNCNS-VD.
Fe-UNCNS-VD was prepared by similar method to FeSAs-UNCNS except without vacuum pumping step.
Synthesis of FeNPs-UNCNS.
FeNPs-UNCNS was prepared by a similar method to FeSAs-UNCNS except using more $Fe(acac)_3$.
Synthesis of UNCNS.
UNCNS was prepared by a similar method to FeSAs-UNCNS except without $Fe(acac)_3$.
Synthesis of ZIF-L.
$Zn(NO_3)_2{\cdot}6H_2O$ (595 mg, 2 mmol) was dissolved in 40 ml deionized water to form solution A. 2-Methylimidazole (1313.5 mg, 16 mmol) was dissolved in 40 ml deionized water to form solution B. Solution A was quickly poured into solution B, and stirred at 25 ^oC for 6 h. The product was obtained after centrifugation, washing and drying step.
Synthesis of Fe-NCNS.
The $Fe(acac)_3$@ZIF-L was prepared by a similar method to $Fe(acac)_3$@U-ZIF-L except using ZIF-L instead of U-ZIF-L. Then $Fe(acac)_3$@ZIF-L was heated to 900 ^oC at a rate of 5 ^oC min^{-1} in argon atmosphere for 180 min to yield Fe-NCNS.
Synthesis of FeSAs-NC.
The FeSAs-NC was synthesized according to a reference.[1] Typically, 2-methylimidazole (1314 mg, 16 mmol) was dissolved in 15 ml methanol with stirring in flask A. $Zn(NO_3)_2{\cdot}6H_2O$ (1190 mg, 4 mmol) and $Fe(acac)_3$ (141 mg, 0.4 mmol) were dissolved in 30 ml methanol under ultrasound for 15 min to form a clear solution in flask B. Then, flask B was subsequently added into flask A with vigorous stirring for 1h at room temperature. The solution was transferred into a 100 ml Teflon-lined stainless-steel autoclave and heated at 120 °C for 4h. The obtained product was separated by centrifugation and washed subsequently with DMF for third and methanol for twice and finally dried at 70 °C under vacuum for overnight.
The power was transferred into a ceramic boat and placed in a tube furnace. The sample was heated to 900 °C with a heating rate of 5 °C min^{-1} and kept at 900 °C for 3h under flowing argon gas and then naturally cooled to room temperature. The obtained material was directly used without further treatment.
Material characterizations.
The morphologies were characterized by a transmission electron microscope (TEM, JEM-2100F) operated at an acceleration voltage of 200 kV and a field emission scanning electron microscope (SEM, Nova NanoSEM 450, FEI). Aberration-corrected high-angle annular dark-field scanning transmission electron microscopy (AC-HAADF-STEM) was carried out on JEM-ARM200F (JEOL, Tokyo, Japan) TEM/STEM operated at 200 kV with cold field emission gun and aberration corrector. The thickness was sequentially examined by Bruker Dimension Icon AFM operated in a tapping mode at a 1.0 Hz scan rate. X-ray photoelectron spectroscope (XPS) was conducted on a Thermo ESCALAB spectrometer using a monochromatic Al Kα radiation (hv=1486.6 eV). The energy calibration of the spectrometer was performed using the C 1s peak at 284.8 eV. The prepared samples were examined by X-ray diffraction (XRD, Bruker D8 ADVANCE), field emission scanning electron microscopy (FEI VERIOS460), and Raman spectrometer (LabRAM HR Evolution). The inductively coupled plasma optical emission spectrometer (ICP-OES) measurements were conducted on an Icap7600 spectrometer for metal elemental analysis. The surface areas were measured by Brunauer-Emmett-Teller

(BET, Micromeritics ASAP 2460) in nitrogen adsorption isotherms at 77 K, using a sample degas system (VacPrep 061) with 8 h outgas at 100°C.

XAFS measurement.

The X-ray absorption find structure spectra (Fe K-edge) were collected at 1W1B station in Beijing Synchrotron Radiation Facility (BSRF). The storage rings of BSRF was operated at 2.5 GeV with a maximum current of 250 Ma. Using Si (111) double-crystal monochromator, the data collection was carried out in transmission mode using ionization chamber for Fe foil, FeO, Fe_2O_3, FePc and in fluorescence excitation mode using a Lytle detector for FeSAs-UNCNS. All spectra were collected in ambient conditions.

Electrochemical measurements for ORR

All the electrochemical measurements were carried out in a conventional three-electrode system on a CHI 660 electrochemical station (Shanghai Chenhua, China) at room temperature. A rotating disk electrode (RDE) with a glassy carbon (GC) disk of 5 mm in diameter was used as the substrate for the working electrode. A calibrated saturated calomel electrode and a graphite rod were used as reference and counter electrode, respectively. 4 mg of catalyst was dispersed in 1 ml of a solution containing 0.685 ml of ethanol, 0.295 ml of water and 20 µl of 5 wt% Nafion solution, and then sonicated for 2 h to form a homogeneous catalyst ink. Then a certain volume of the catalyst ink was pipetted onto the GC surface with the nonprecious catalyst loading 0.5 mg cm^{-2} and the loading of Pt/C 0.1019 mg cm^{-2}. Before tests, O_2 flow was carried out through the electrolyte in the cell for about 30 min to achieve the O_2 saturated solution. The cyclic voltammetry (CV) tests were measured in an O_2-saturated 0.1 M KOH solution with a scan rate of 50 mV s^{-1}. RDE tests were conducted in O_2-saturated 0.1 M KOH at different rotation rates with a sweep rate of 10 mV s^{-1} at room temperature. In this work was referred versus the reversible hydrogen electrode (RHE):

$$E_{RHE} = E_{SCE} + 0.241 + 0.0591pH$$

The electron transfer number (n) were determined by the Koutecky-Levich equation:

$$\frac{1}{J} = \frac{1}{J_L} + \frac{1}{J_K} = \frac{1}{B\omega^{\frac{1}{2}}} + \frac{1}{J_K}$$

$$B = 0.62nFC_0D_0^{\frac{2}{3}}V^{-\frac{1}{6}}$$

where J, J_K, and J_L are the measured, kinetic and limiting current density respectively, ω is the angular velocity of the disk, n is the overall number of electrons transferred in oxygen reduction, F is the Faraday constant (96485 C mol^{-1}), C_0 is the bulk concentration of O_2 (1.2×10^{-6} mol cm^{-3}), D_0 is the diffusion coefficient of O_2 in 0.1 M KOH (1.9×10^{-5} cm^2 s^{-1}), and V is the kinematic viscosity of the electrolyte (0.01 cm^2 s^{-1}), and k is the electron transfer rate constant.

The rotating ring-disk electrode (RRDE) examinations were carried out with the Pt ring electrode to test the ring current (I_{ring}). The hydrogen peroxide yield (H_2O_2 (%)) and electron transfer number (n) were calculated based on the following equations:

$$H_2O_2(\%) = 200 \times \frac{I_{ring}}{\left(\frac{I_{ring}}{N}\right) + I_{disk}}$$

$$n = 4 \times \frac{I_{disk}}{\frac{I_{ring}}{N} + I_{disk}}$$

where I_{disk} is the disk current, I_{ring} is the ring current and N = 0.37 is the current collection efficiency of the platinum ring. The disk electrode was scanned negatively (50 mV s^{-1}). Meanwhile, a high potential (1.20 V vs RHE) was applied on the ring electrode, leading to the electro-oxidation of H_2O_2, which occurred during the oxygen reduction process.

The double-layer capacitance (C_{dl}) is used to estimate the electrochemically active surface area (ECSA) of the catalyst. It is obtained by testing the CV of the non-Faraday voltage range at different scan rates in 0.1 M KOH.

The ECSA was determined based on the following equation:

$$ECSA = \frac{C_{dI}}{C_s}$$

where C_{dl} is the double-layer capacitance value which can be estimated from the linear slope of the fitting line of capacitive currents versus scan rates, and the C_s value is adopted as 0.04 mF cm^{-2}.

Quantification of the active sites.

The site density (SD) was obtained according to the method presented by Kucernak et al.[2] Briefly, extensive cycling in pH=5.2 acetate buffer alternatively in O_2 and N_2 was performed to obtain nonchanging cyclic voltammetry curves in N_2. Then the catalyst was poisoned by $NaNO_2$. The ORR performance was recorded before, during and after the nitrite absorption. Nitrite stripping was conducted in the region of 0.4 to -0.55 V vs RHE. The excess in cathodic charge (Q_{strip}) was proportional to the SD, and the SD was calculated by Q_{strip}:

$$Q_{strip}(C \cdot g^{-1}) = \frac{S_{Stripping\ CV} - S_{baseine\ CV}}{V_{scan}\ (V \cdot S^{-1})\ \times L_C(mg \cdot cm^{-2})}$$

$$SD(moL\cdot g^{-1})\frac{Q_{Strip}(C\cdot g^{-1})}{n_{strip}\times F(C\cdot moL)}$$

where Q_{strip} ($C\cdot g^{-1}$) is the excess Coulometric charge associated with the stripping peak, the F is the Faraday constant (F = 96485 C mol^{-1}), n_{strip} (=5) is the number of electrons associated with the reduction of one nitrite per site, L_C is the catalyst loading (0.27 mg cm^{-2}).

Zn-air battery measurements.

10 mg catalyst was dispersed in 1 ml solution containing 470 μI ethanol, 470 μl H_2O and 60 μl 5% Nafion solution. Then, the slurry was sprayed on the hydrophobic carbon paper uniformly. This carbon paper was used as the air cathode while a polished Zn foil was used as the anode. For the primary Zn-air batteries, 6 mol L^{-1} KOH was used as electrolyte and the catalyst loading was 2 mg cm^{-2}. In addition, 0.54 M NaCl was added as an electrolyte for the seawater-based Zn-air battery. For the rechargeable Zn-air battery, 0.2 mol L^{-1} zinc acetate dehydrated electrolyte was added. The catalyst was loaded with 1 mg cm^{-2}. Batteries were tested on CHI 660E electrochemical workstation at room temperature. The charge-discharge cycle stability for rechargeable Zn-air battery was carried out with a cycling interval of 20 min (10 min for charging and 10 min for discharging).

Computational details.

Density functional theory (DFT) calculations was carried out using the Vienna ab-initio simulation package (VASP) with projector augmented wave pseudo-potentials (PAW) to describe the interaction between atomic cores and valence electrons.[3] The Perdew-Burke-Ernzerhof (PBE) functional within the generalized gradient approximation (GGA) were used to implement DFT calculations.[4] The DFT+U method was used to correct the Coulomb repulsion of 3d electrons of transition metal, and the effective U value of 3d electrons of Fe was set to 5.3 eV. Vander Waals effects were applied using Grimme's DFT-D_3 correction method, and dipole corrections along the surface normal were considered. All calculations were spin-polarized. The wave functions of valence electrons were expanded using a plane wave basis set with a cut-off energy of 450 eV. The convergence criteria for energy and force during geometrical optimization were set to 10^{-5} eV and 0.02 eV $Å^{-1}$, respectively. A vacuum space of 15 Å was applied to avoid interactions along the z-direction. Brillouin zone sampling was conducted using a Monkhorst-Pack grid with 2 × 2 × 1 k-point mesh. To module the Fe-N_4O active site of FeSAs-UNCNS electrocatalyst, we selected double-layer graphene as the carbon support, in which the top graphene layer supported the FeN_4 and in the second layer of graphene O elements were introduced. For comparison, an Fe-N_4 in the graphene layer was built to model a typical Fe-N_4 sites by pyrolysis method and an isolated octahedral Fe_6 cluster was also built to model the Fe nanoparticles. To module the FeN_4 electrocatalyst, a Fe-N_4 site is embedded in a periodic 4×4 graphene support (32 carbon sites) with lattice parameters a=b=9.84 Å and γ=120°. The Fe-N_4O model is based on the FeN_4 model, with the addition of a second layer of graphene structure and the introduction of N and O elements. It involves an O atom bridging the Fe-N_4 sites in the first layer. To compare the electrocatalytic reactivity between Fe-N_4O and Fe-N_4 site and Fe particles, an isolated octahedral Fe_6 cluster was also built to model the Fe particles. The four-electron pathway by which the ORR occurs under base condition are generally reported to proceed according to the following steps

(1) $O_2(g) + * \rightarrow *O_2$

(2) $*O_2 + H_2O(l) + e^- \rightarrow *OOH + OH^-$

(3) $*OOH + e^- \rightarrow *O + OH^-$

(4) $*O + H_2O(l) + e- \rightarrow *OH + OH^-$

(5) $*OH + e^- \rightarrow OH^- + *$

where * represents a surface site. Here, we applied a method previously developed for modeling the thermochemistry of electrochemical reactions based on density functional calculations.[5] In this method, the Gibbs free energy is used as a descriptor to evaluate whether reactions proceed spontaneously. Specifically, the Gibbs free energy can be obtained by adding corrections including entropic (TS) and zero-point energy (ZPE) contributions to the calculated DFT energy, so that

(7) $\Delta G = \Delta E_{DFT} + \Delta ZPE - T\Delta S - eU$

where ΔE_{DFT} is the calculated DFT reaction energy, ΔZPE is the change in ZPE calculated from the vibrational frequencies and ΔS is the change in the entropy taken from thermodynamics databases. In the current work, electrode potentials are reported with respect to the reversible hydrogen electrode, which makes the standard electrochemical potential of an electron involved in a reaction (G_e) equal to -eU, and the standard electrochemical potential of the proton (G_{H+}) equal to that of the hydrogen atom in gaseous H_2 ($1/2G_{H2}$). Considering that the triplet state of the O_2 molecule is poorly described in the current DFT scheme, the free energy of the O_2 molecule was derived according to $G_{O2} = 2G_{H2O} - 2G_{H2} + 4.92$ eV. The adsorption free energy of various oxygenated species could therefore be described by the equations below:

(8) $\Delta G_{*OOH} = G_{*OOH} + 3/2G_{H2} - G* - 2G_{H2O}$

(9) $\Delta G_{*O} = G_{*O} + G_{H2} - G* - G_{H2O}$

(10) $\Delta G_{*OH} = G_{*OH} + 1/2G_{H2} - G* - G_{H2O}$

Thus, the reaction free energies of equations (8)-(10) can be determined from the adsorption free energies of the oxygen-containing intermediates formed during ORR:

(11) $\Delta G_1 = 4.92 - \Delta G_{*OOH} - eU$

(12) $\Delta G_2 = \Delta G_{*OOH} - \Delta G_{*O} - eU$
(13) $\Delta G_3 = \Delta G_{*O} - \Delta G_{*OH} - eU$
(14) $\Delta G_4 = \Delta G_{*OH} - eU$
Since the reaction free energy of the whole ORR process is described in these equations, free energy diagrams can be constructed for ORR on the different catalysts (i.e., reaction progress diagrams) by assigning the absolute free energy of the first stage of ORR to zero.

Figures and Tables

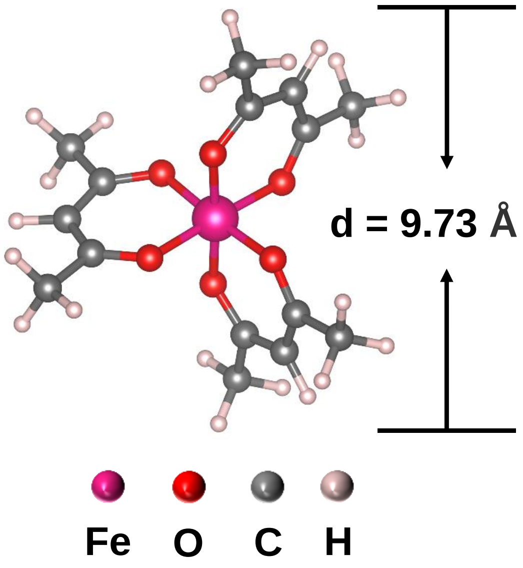

Figure S1. The molecular structure of Fe(acac)$_3$.

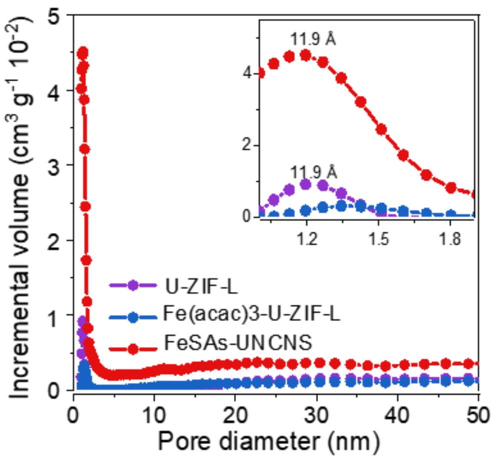

Figure S2. The pore size distributions of U-ZIF-L, Fe(acac)$_3$@U-ZIF-L, and FeSAs-UNCNS.

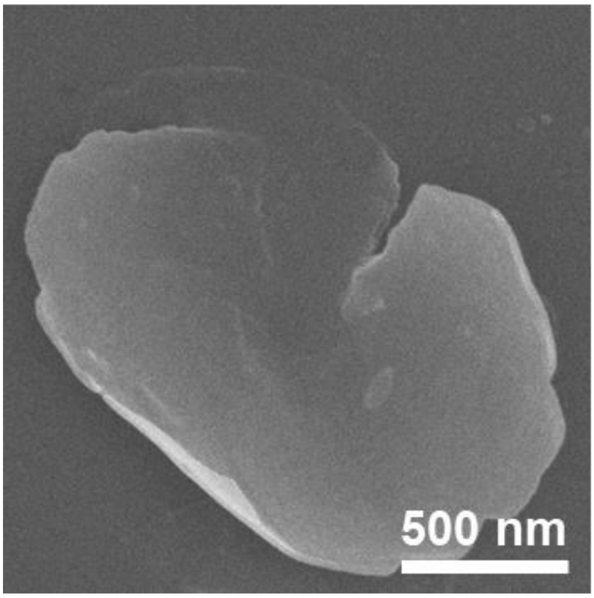

Figure S3. The SEM image of U-ZIF-L.

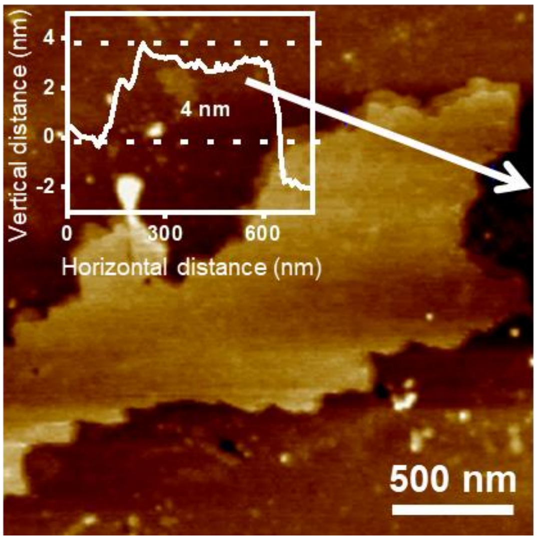

Figure S4. The AFM image of U-ZIF-L.

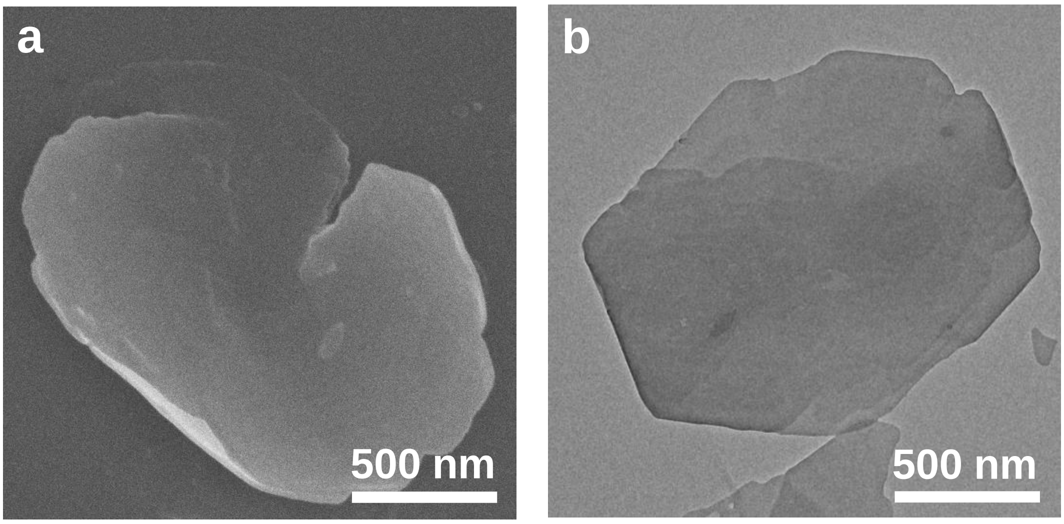

Figure S5. (a) SEM and (b) TEM images of $Fe(acac)_3$@U-ZIF-L.

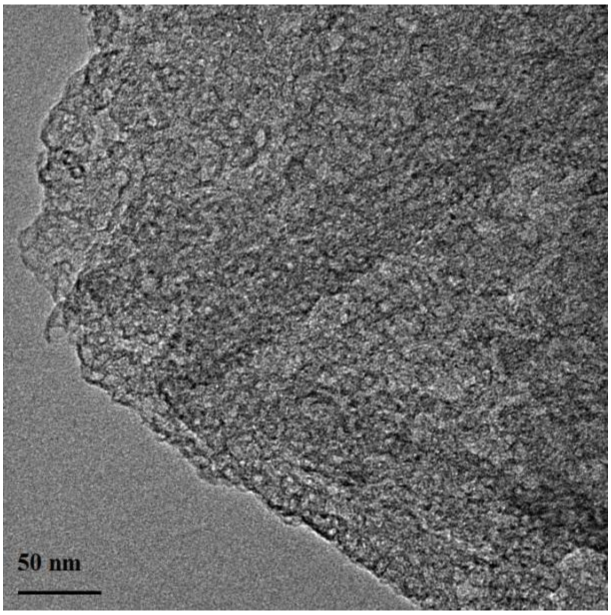

Figure S6. The HRTEM image of FeSAs-UNCNS.

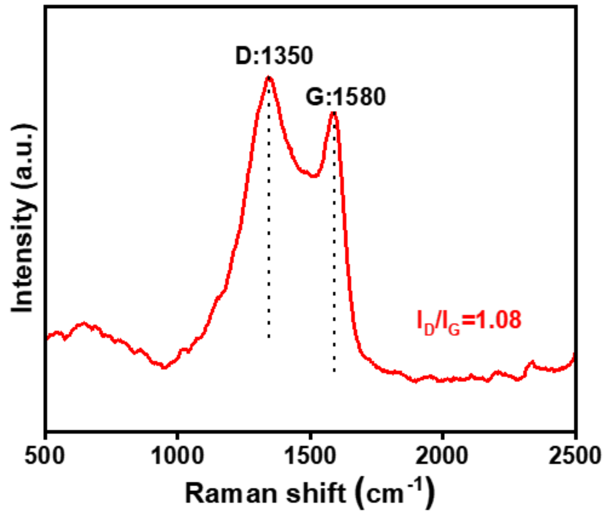

Figure S7. Raman spectrum of the FeSAs-UNCNS.

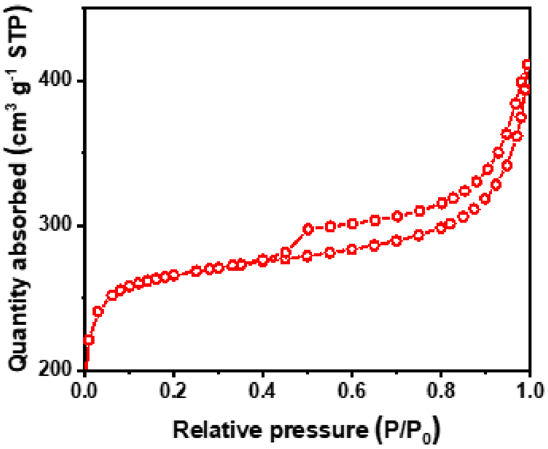

Figure S8. N_2 adsorption/desorption isotherms of FeSAs-UNCNS.

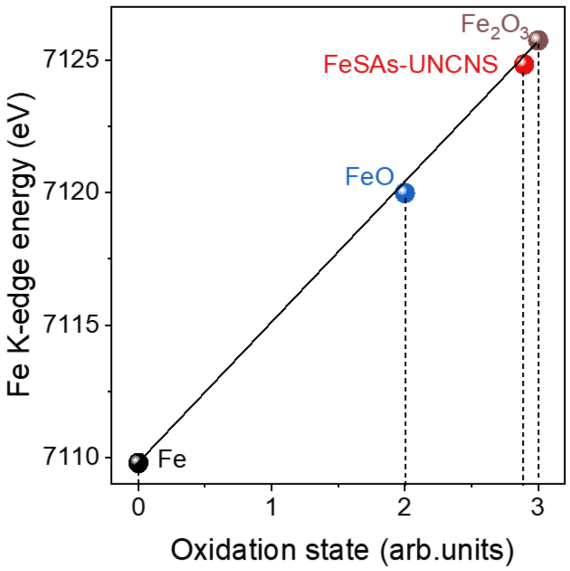

Figure S9. The average oxidation states of Fe from XANES spectra.

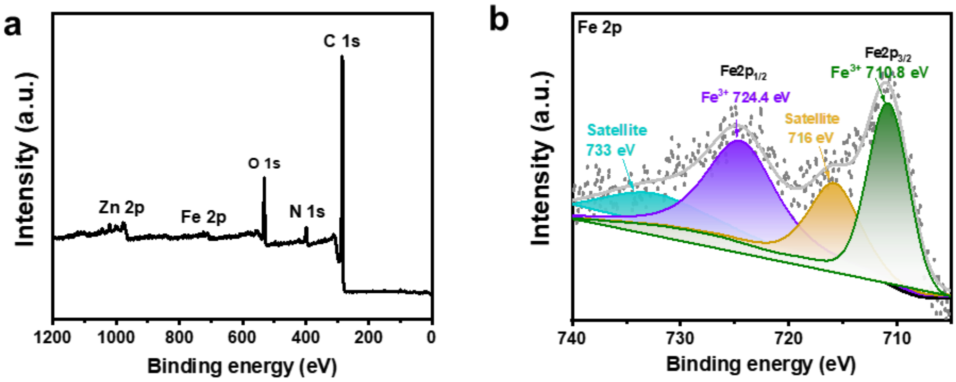

Figure S10. XPS analysis of FeSAs-UNCNS. (a) XPS survey spectrum. (b) Fe 2p spectrum.

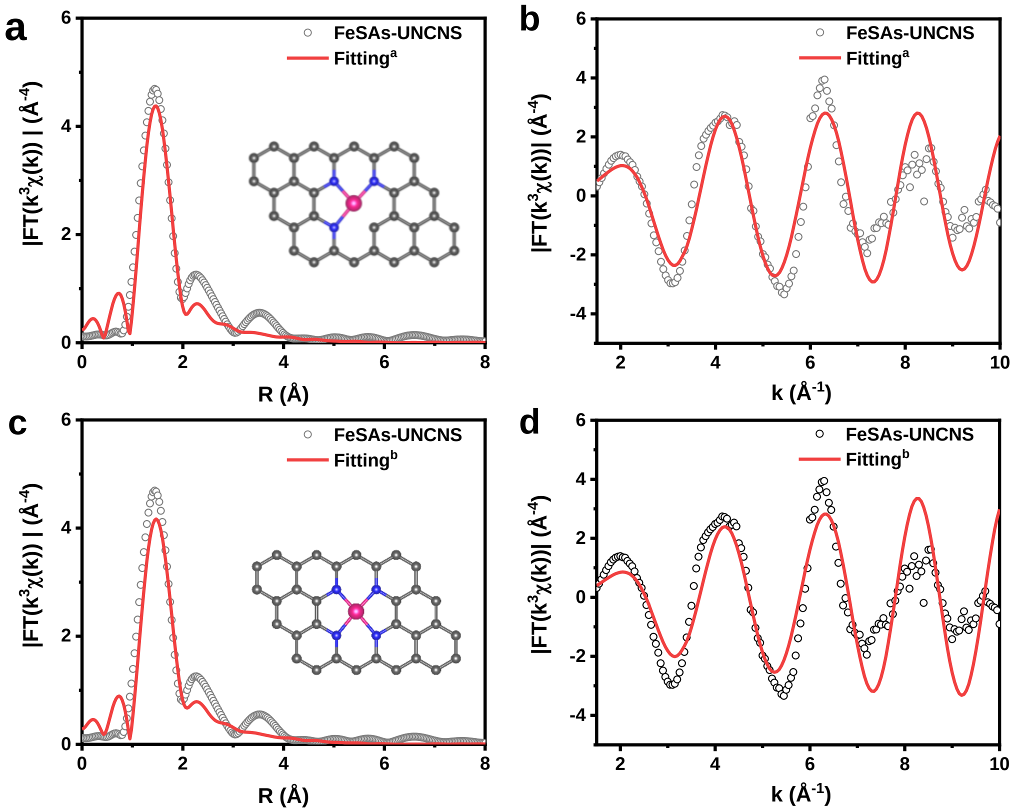

Figure S11. Different model fitting. (a, c) The EXAFS fitting curve at R space and schematic model of FeSAs-UNCNS. (b, d) The EXAFS fitting curve at k space of FeSAs-UNCNS. [a]Fe-N_3, [b]Fe-N_4.

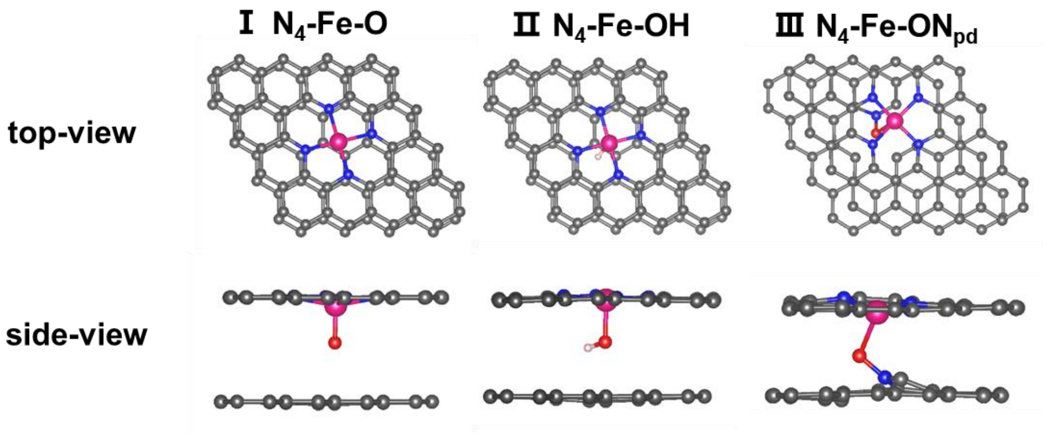

Figure S12. Optimized configurations for different Fe-N_4O structures (Fe: pink, N: blue, O: red, and C: gray).

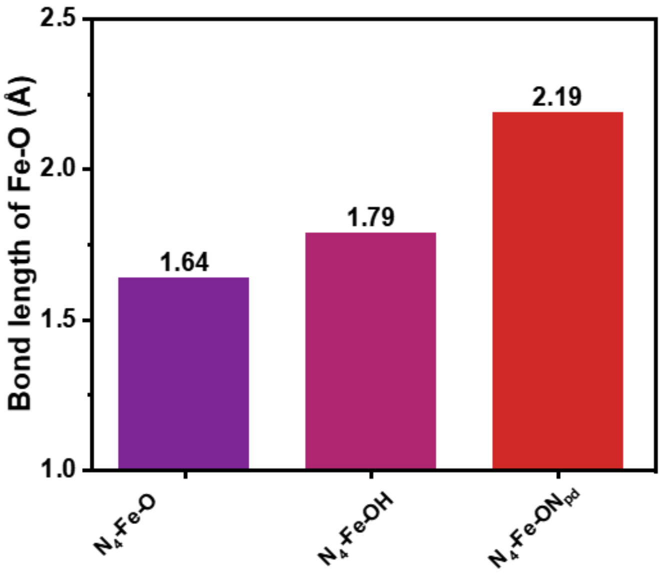

Figure S13. Fe-O bond lengths for the proposed Fe-N_4O structures.

Notes: Based on the EXAFS fitting result, three possible structure models with one Fe atom coordinated by four N atoms and one O atom were proposed (Fig. S12). These configurations were optimized by DFT calculations, and the Fe-O bond lengths were used to screen out the reasonable model (Fig. S13). Since the Fe-O bond distance was 2.10 Å in the EXAFS fitting parameters, the models with Fe-O bond length <2.0 Å (i. e. N_4-Fe-O, N_4-Fe-OH) were ruled out. The most satisfactory model was N_4-Fe-ON_{pd}, where one Fe atom was coordinated by four N atoms in the same plane and one axial O atom located between the two graphene layers and bridged these layers through bonding to the pyridinic N atom from the second layer.

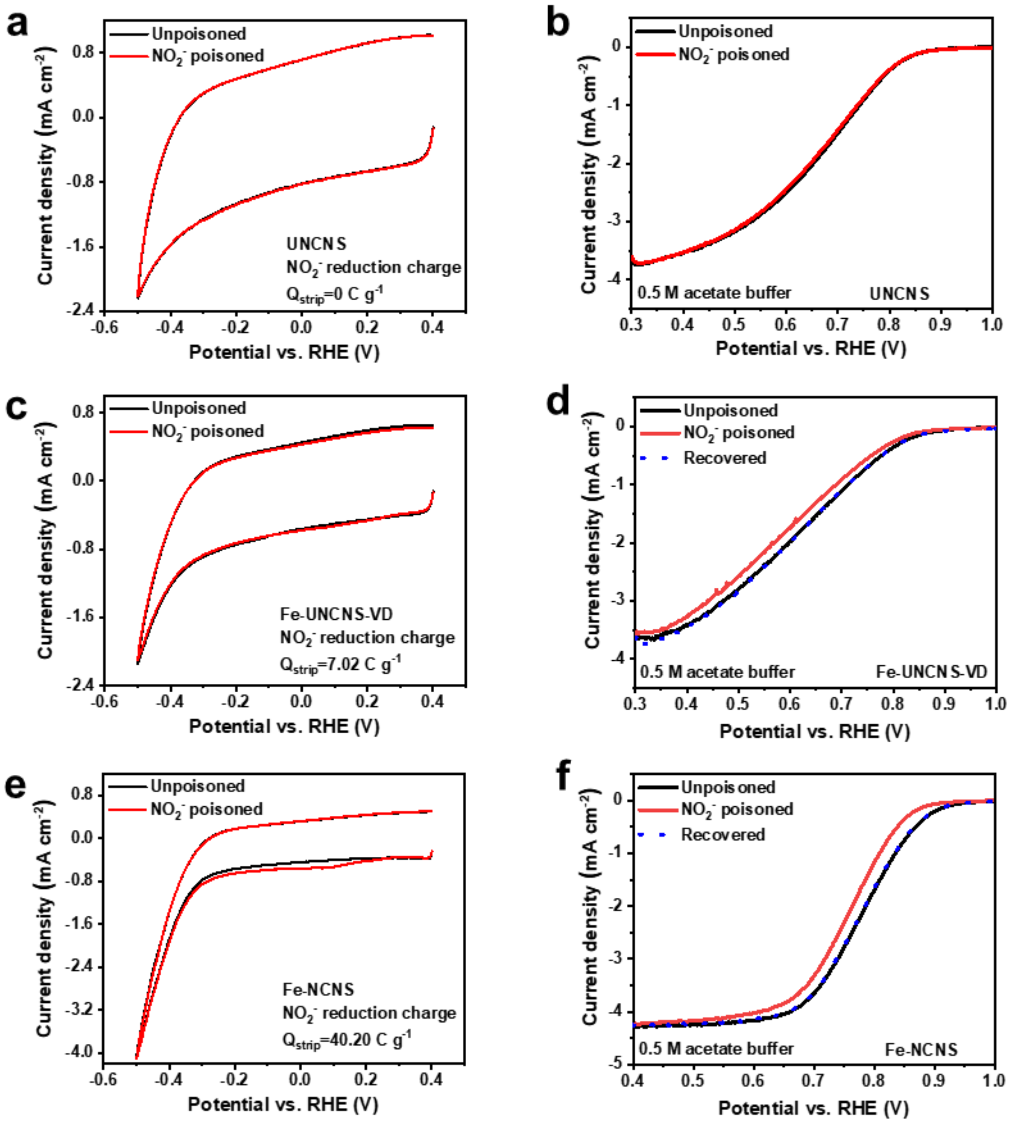

Figure S14. Determination of the site density (SD) of Fe-N_4 for UNCNS (a, b), Fe-UNCNS-VD (c, d), Fe-NCNS (e, f). (a, c, e) ORR LSV curves before, during and after nitrite adsorption in an O_2-saturated 0.5 M acetate buffer at pH 5.2. (b, d, f) CV curves before and during nitrite adsorption in the nitrite reductive stripping region. Catalyst loading: 0.27 mg cm^{-2}.

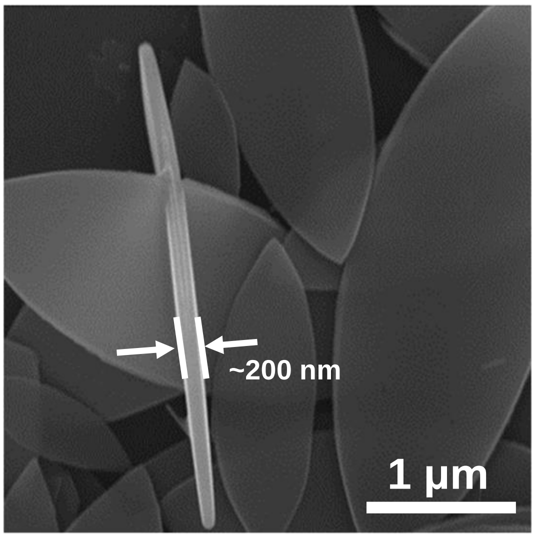

Figure S15. SEM image of ZIF-L.

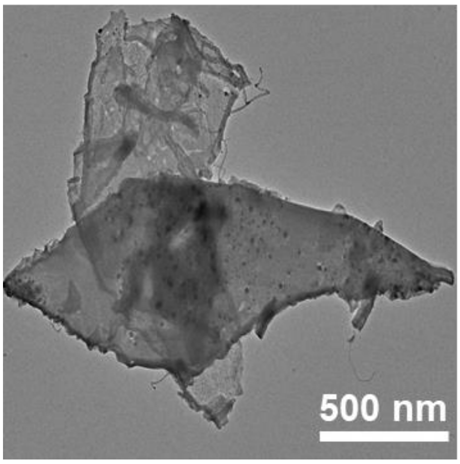

Figure S16. TEM image of Fe-NCNS.

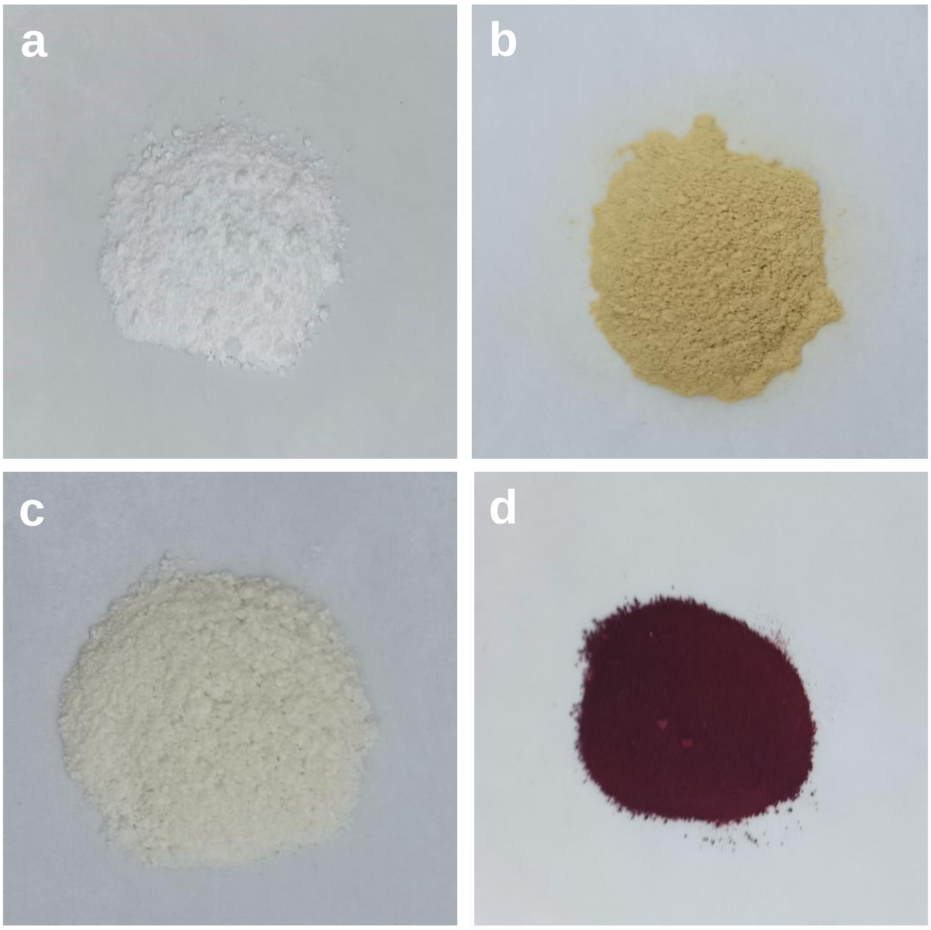

Figure S17. Photographs of (a) U-ZIF-L, (b) $Fe(acac)_3$@U-ZIF-L, (c) $Fe(acac)_3$@U-ZIF-L-VD, (d) $Fe(acac)_3$.

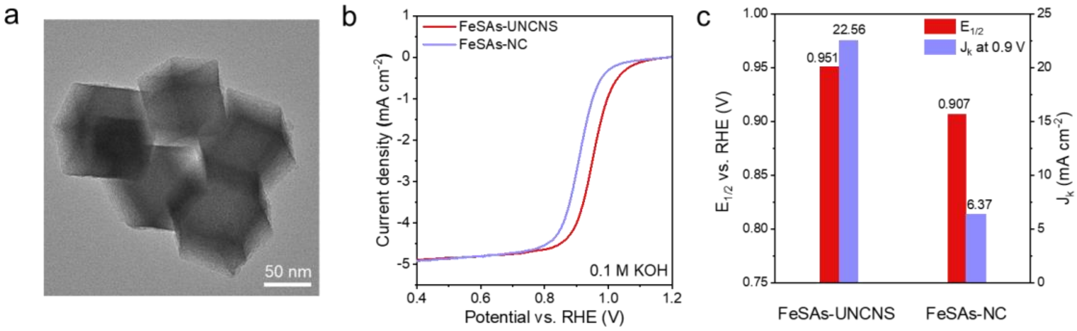

Figure S18. The TEM, LSV curve and J_k and $E_{1/2}$ of FeSAs-NC.

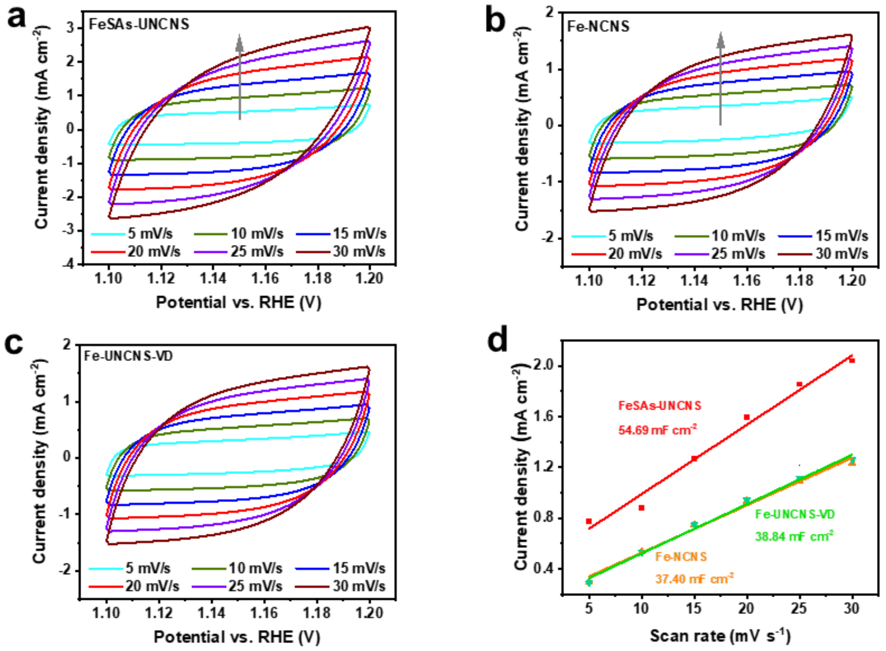

Figure S19. CV curves of (a) FeSAs-UNCNS, (b) Fe-NCNS and (c) Fe-UNCNS-VD in 0.1 M KOH solution in the region of 1.11~1.20 V vs. RHE at scan rate of 5, 10, 15, 20, 25 and 30 mV s^{-1} without Faradaic process. (d) Average variance between the anodic and cathodic current at 1.05 V vs. RHE against the scan rate.

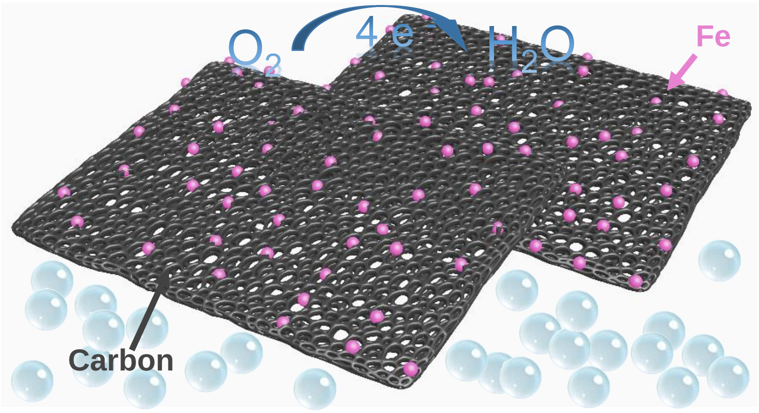

Figure S20. Illustration of ORR on FeSAs-UNCNS.

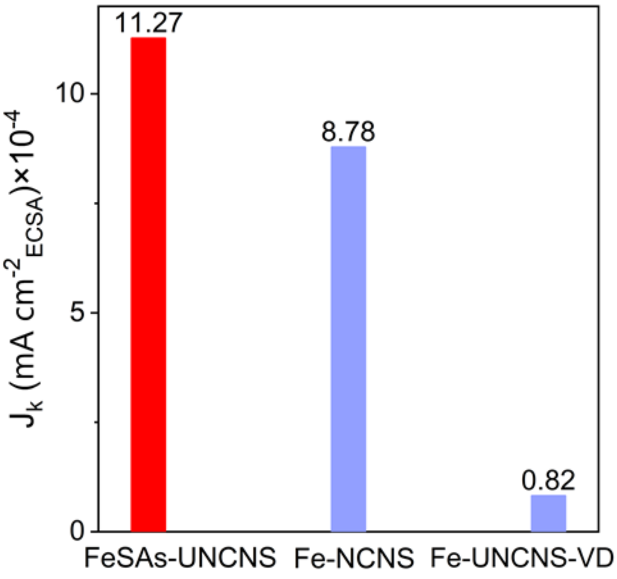

Figure S21. The kinetic current density normalized by ECSA for FeSAs-UNCNS, Fe-NCNS and Fe-UNCNS-VD.

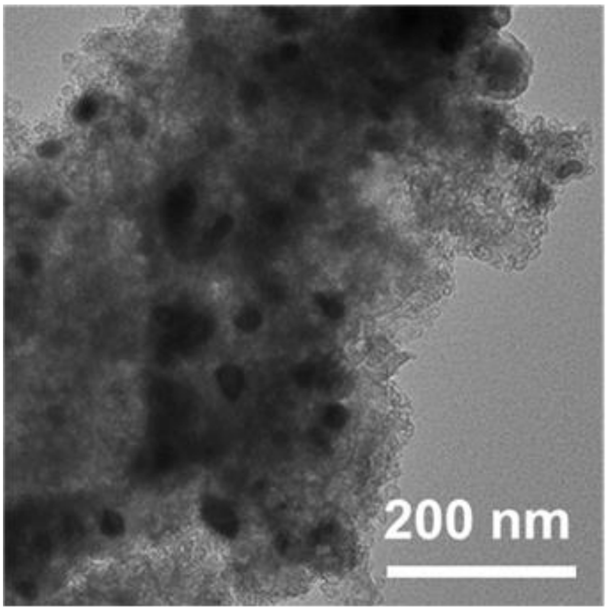

Figure S22. TEM image of FeNPs-UNCNS.

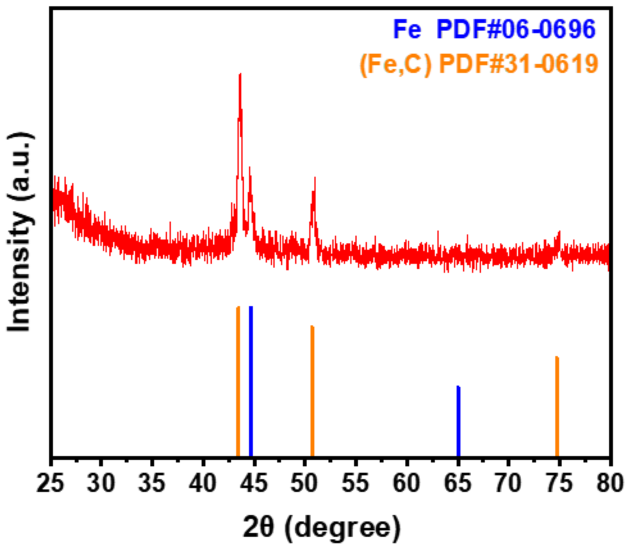

Figure S23. XRD pattern of FeNPs-UNCNS.

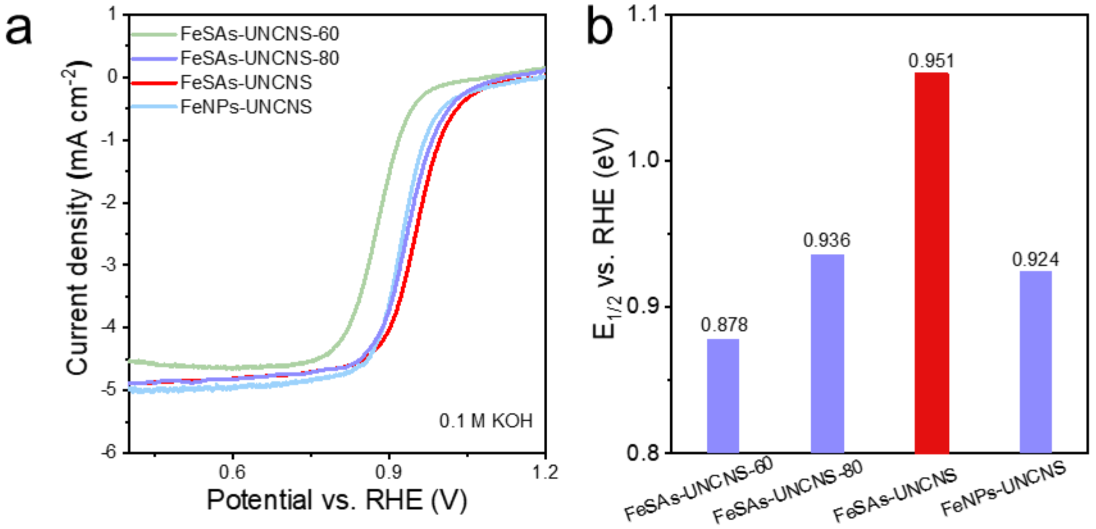

Figure S24. LSV curves of UNCNS supported iron catalysts prepared using different amounts of $Fe(acac)_3$.

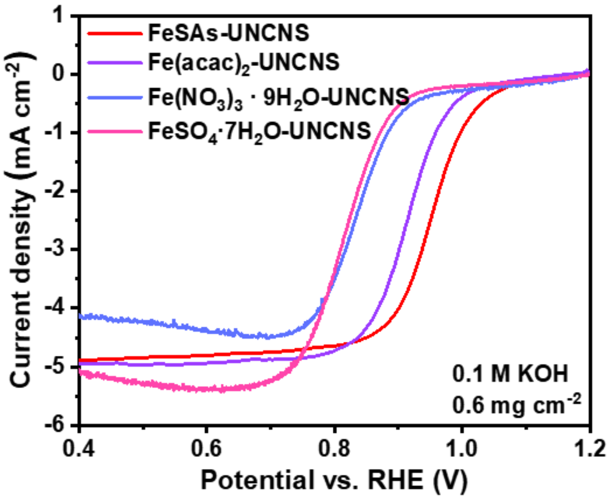

Figure S25. LSV curves of various Fe electrocatalysts in an O_2-saturated 0.1 M KOH solution at a rotation speed of 1,600 rpm and a sweep rate of 10 mV s^{-1}.

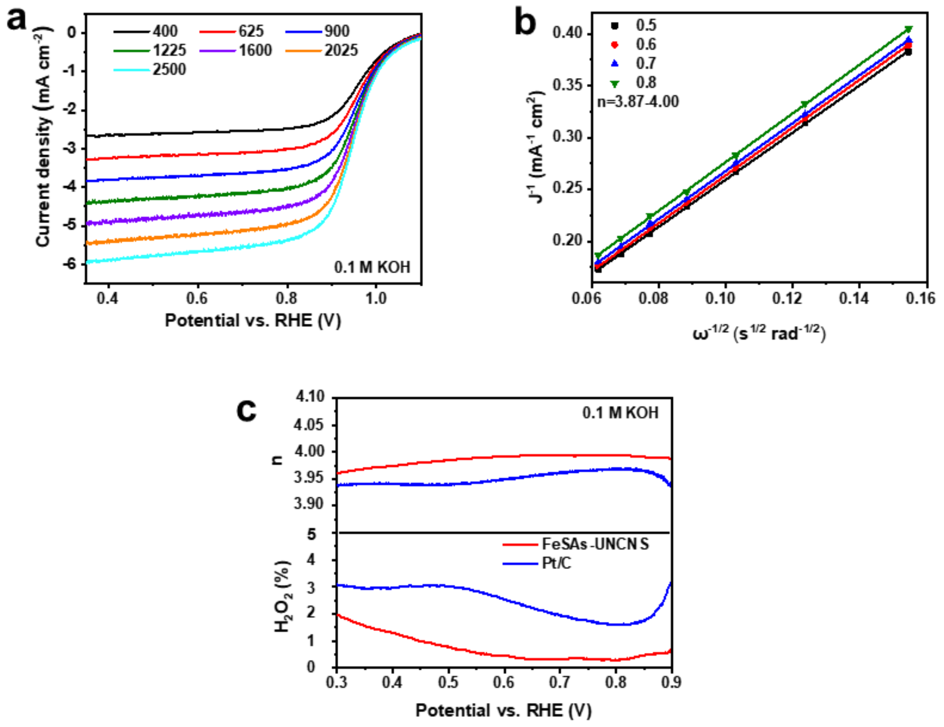

Figure S26 (a) LSV curves of FeSAs-UNCNS for ORR recorded at different rotating speeds. (b) K-L plots of FeSAs-UNCNS in the potential range from 0.5 V to 0.8 V vs. RHE. (c) Electron-transfer number and H_2O_2 yield versus the potential for FeSAs-UNCNS and Pt/C.

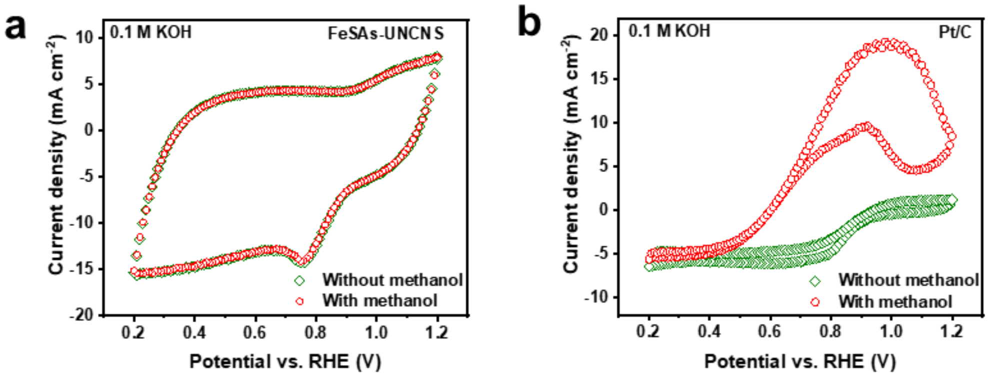

Figure S27. (a) Cyclic voltammetry (CV) data for FeSAs-UNCNS in O_2-saturated 0.1 M KOH without and with 1.0 M CH_3OH. (b) Cyclic voltammetry (CV) data for Pt/C in O_2-saturated 0.1 M KOH without and with 1.0 M CH_3OH.

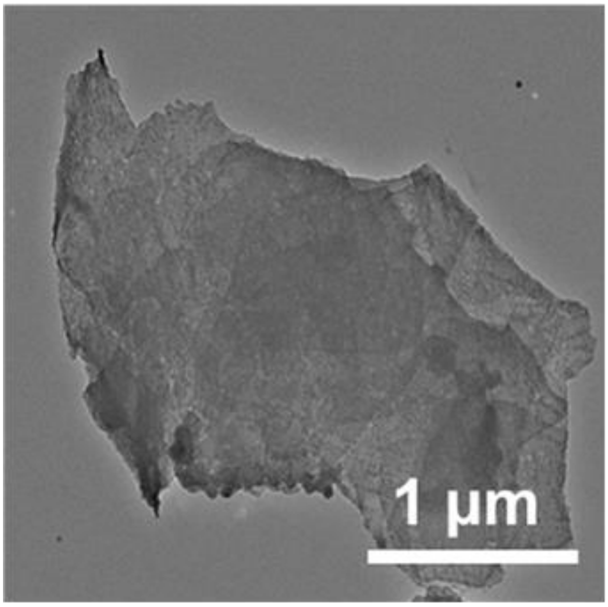

Figure S28. TEM image of FeSAs-UNCNS after 5000 cycles.

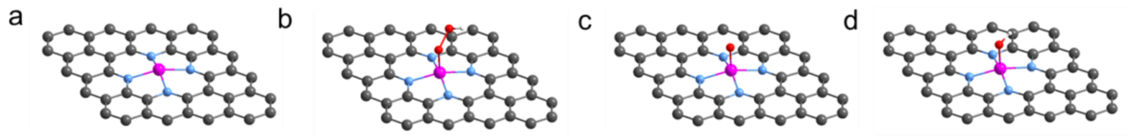

Figure S29. (a) Computational models of Fe-N_4. Configurations of (b) *OOH (c) *O (d) *OH on Fe-N_4.

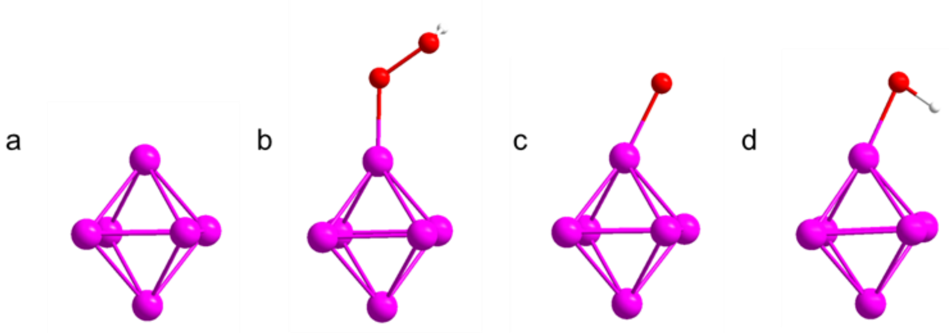

Figure S30. (a) Computational models of Fe_6 particle. Configurations of (b) *OOH (c) *O (d) *OH on Fe_6 particle.

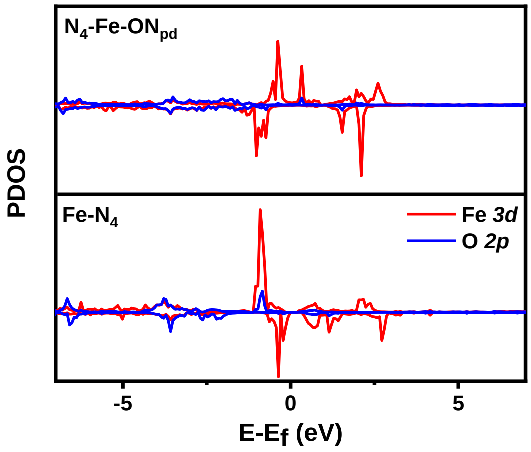

Figure S31. PDOS of structures N_4-Fe-ON_{pd} and Fe-N_4 after adsorption of *OH.

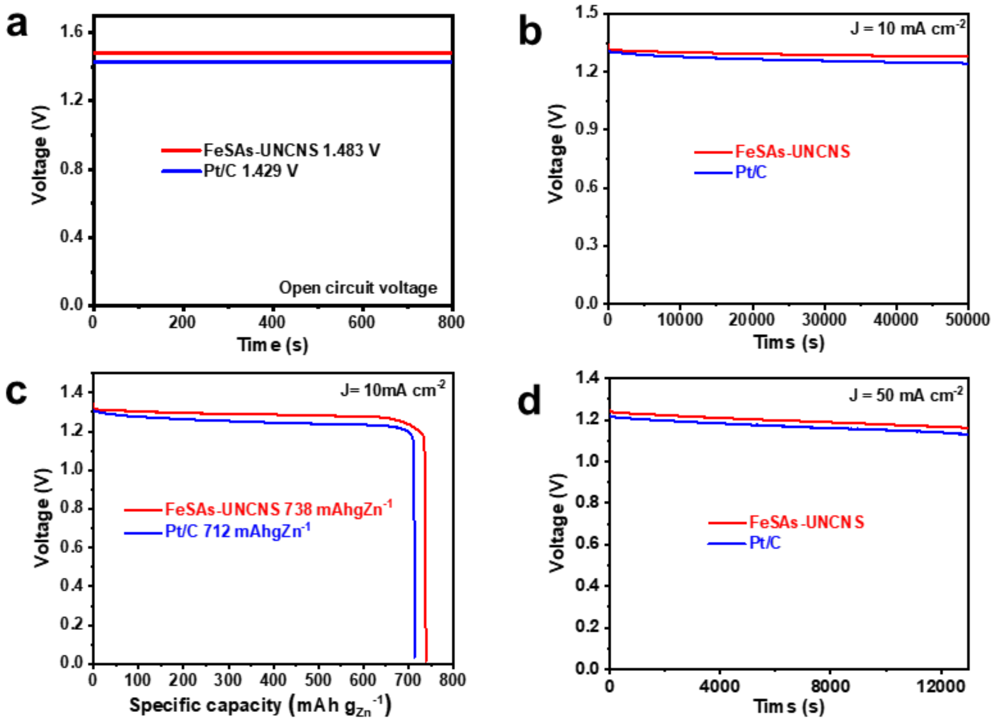

Figure S32. Primary Zn-air battery performance. (a) open-circuit voltage measurements for FeSAs-UNCNS and Pt/C cathode. (b, d) Long-time discharge curves of primary batteries using FeSAs-UNCNS and Pt/C as the cathode catalysts at the current density of 10 mA cm^{-2} and 50 mA cm^{-2}. (c) Long-time galvanostatic discharge curves of Zn-air batteries with FeSAs-UNCNS and Pt/C cathodes at 10 mA cm^{-2}.

Table S1. Nitrogen adsorption-desorption results, BET surface area and the pore volume of U-ZIF-L, $Fe(acac)_3$@U-ZIF-L and FeSAs-UNCNS.

**Sample**	**U-ZIF-L**	**$Fe(acac)_3$@U-ZIF-L**	**FeSAs-UNCNS**
BET surface area ($m^2\ g^{-1}$)	123.8	59.0	1017.8
Volume of total pore ($cm^3\ g^{-1}$)	0.11	0.085	0.64
Volume of micropores ($cm^3\ g^{-1}$)	0.048	0.018	0.35
Volume of mesoporous and macroporous ($cm^3\ g^{-1}$)	0.062	0.067	0.29

Table S2. Fe and Zn contents determined by ICP-OES.

**Sample**	**Fe Content (wt%)**	**Zn Content (wt%)**	**SD (10^{19} sites g^{-1}) by NO_2^-**
FeSAs-UNCNS	4.22	0.08	11.1
Fe-NCNS	3.54	1.39	5.02
Fe-UNCNS-VD	0.09	5.15	0.88
FeSAs-NC	2.42	1.54	8.03
UNCNS	-	6.96	-

Table S3. EXAFS fitting parameters at the Fe K-edge. (S_0^2=0.72)

Sample	Path	C.N.	R (Å)	σ^2×103(Å^2)	ΔE (eV)	R factor
Fe foil	Fe-Fe	8*	2.47±0.01	5.0±0.8	6.6±1.2	0.001
	Fe-Fe	6*	2.84±0.01	6.2±1.6	5.0±2.5	
FeSAs-UNCNS	Fe-N	3.8±0.2	2.01±0.01	5.1±5.2	-1.9±2.3	0.009
	Fe-O	1.2±0.4	2.10±0.21			

C.N: coordination numbers; R: bond distance; σ^2: Debye-Waller factors; ΔE: the inner potential correction. R factor: goodness of fit. * fitting with fixed parameter.

Table S4. Performance comparison for ORR catalysts.

	Catalyst	$E_{1/2}$(V)	Electrolyte	SD (10^{19} sites g^{-1}) by NO_2^-	Reference
alkaline	**FeSAs-UNCNS**	**0.951**	**0.1 M KOH**	**11.1**	**This work**
	OAC	0.86	0.1 M KOH	2.58	Appl. Catal. B-Environ. **2022**, 305, 121058.
	Bz-Fe_1-UNCNS	0.94	0.1 M KOH	1.601	Chem. Eng. J. **2021**, 424, 130401.
acidic	Fe-UNCNS $^{\Delta\text{-DCDA}}$	0.80	0.5 M H_2SO_4	4.7	Nat. Catal. **2022**, 5, 311.
	FeNC-CVD-750	0.85	0.5 M H_2SO_4	6.0	Nat. Mater. **2021**, 20, 1385-139.
	TPI(10%)@Z8(SiO_2)-650-C	0.82	0.5 M H_2SO_4	3.42	Nat. Catal. **2019**, 2, 259.
	$FPHEN_3$	0.748	0.5 M H_2SO_4	0.424	Electrochim. Acta **2021**, 394, 139105.
	Z8@DA-FIP-950-C	0.828	0.1 M $HClO_4$	3.79	Appl. Catal. B-Environ. **2022**, 302, 120860.
N.A.	Fe-N/C	N.A.	N.A.	0.72	Nat. Commun. **2016**, 7, 13285.

Table S5. C_{dl} and ECSA of FeSAs-UNCNS, Fe-NCNS and Fe-UNCNS-VD.

Sample	C_{dl} (mF cm^{-2})	ECSA (cm^{2})
FeSAs-UNCNS	54.69	1417
Fe-NCNS	37.40	935
Fe-UNCNS-VD	38.84	971

Table S6. Fe contents of UNCNS supported iron catalysts.

Sample	$Fe(acac)_3$ (mg)	Fe Content (wt%)
FeSAs-UNCNS-60	60	3.22
FeSAs-UNCNS-80	80	3.63
FeSAs-UNCNS	100	4.22
FeNPs-UNCNS	140	4.31

Table S7. Adsorption free energies for *OOH, *O, and *OH (ΔG_{*OOH}, ΔG_{*O}, and ΔG_{*OH}) on the Fe-N_4 model and Fe-N_4O structure.

Active site	ΔG_{*OOH} (eV)	ΔG_{*O} (eV)	ΔG_{*OH} (eV)
Fe-N_4O	3.86	1.70	0.66
Fe-N_4	3.38	1.28	0.46
FeNPs	2.47	0.91	-0.75

Table S8. The Bader charges of Fe in SACs before and after *OH adsorption. The positive values represent loss of electrons from metals.

**Active site**	**Fe**	**Fe(*OH)**
Fe-N_4O	0.96	1.23
Fe-N_4	1.15	1.3

Table S9. Performance comparison of Zn-air batteries.

Catalyst	Catalyst loading (mg cm^{-2})	Electrolyte	Power Density (mA cm^{-2})	Reference
**FeSAs-UNCNS**	**2**	**6 M KOH**	**306**	**This work**
Mn/C-NO	2	6 M KOH	170	Adv. Mater. **2018**, 30, 1801732.
Fe, Ni-SAs/DNSC	2.3	6 M KOH+0.2 M $Zn(Ac)_2$	160	Chem. Eng. J. **2021**, 426, 130758.
SA-Fe-NHPC	1	6 M KOH+0.2 M $Zn(Ac)_2$	266.4	Adv. Mater. **2020**, 32, 1907399.
Fe-SAs/NPS-HC	1	6 M KOH+0.2 M $Zn(Ac)_2$	195	Nat. Commun. **2018**, 9, 5422.
Fe SAC-MIL101-1000	1	6 M KOH+0.2 M $Zn(Ac)_2$	192.3	Adv. Mater. **2021**, 33, 2101038.
Zn-N_4-O	1	6 M KOH+0.2 M $Zn(Ac)_2$	182	Adv. Sci. **2023**, 10, 2302152.
NBCNT-10	1	6 M KOH	173.9	Chem. Eng. J. **2021**, 422, 130134.
Fe-SA-UNCNS	1	6 M KOH+0.2 M $Zn(Ac)_2$	136.4	J. Mater. Chem. A **2021**, 9, 15919.

Table S10. Summary of the rechargeability and energy efficiencies of the fabricated Zn-air batteries.

**Sample**	**Current density (mA cm^{-2})**	**First cycle energy efficiency (%)**	**Last cycle energy efficiency (%)**	**△V of charge and dischaege (V)**	**Time /Cycles**	**Reference**
FeSAs-UNCNS	5	57.54	55.83	<0.86	180 h/540	This Work
FeP/Fe_2O_3@ NPCA	5	56.71	54.23	<0.888	160 h/960	Adv. Mater. **2020**, 32, 2002292.
Fe-N-GDY	5	-	-	<0.90	-/700	Angew. Chem. Int. Ed. **2022**, 61, e202208238.
Fe_2/Co_1GNCL	-	-	-	<0.8	83.3 h/500	Angew. Chem. Int. Ed. **2020**, 59, 16013.
Co-N/C+NG	5	-	-	<1.07	75 h/450	Small **2022**, 18, 2105329.
ZOMC	5	-	-	<0.76	160 h/500	Adv. Mater. **2020**, 32, 2002170.
Co_3HITP_2	5	-	53.9	-	83.3 h/500	Angew. Chem. Int. Ed. **2020**, 59, 286.
S, N-FeN-C-CNT	5	-	-	-	16.7 h/100	Angew. Chem. Int. Ed. **2017**, 56, 610.
$CoFe_2O$@CC	5	-	-	-	133.3 h/400	Adv. Mater. **2019**, 31, 1904689.
SA-Ir/NC	5	54.54	-	1	100 h/300	Adv. Funct. Mater. **2021**, 31, 2101193.
NGM-Co	5	48.86	-	<1.12	16 h/20	Adv. Mater. **2017**, 29, 1703185.

References

1. Y. Chen, S. Ji, Y. Wang, J. Dong, W. Chen, Z. Li, R. Shen, L. Zheng, Z. Zhuang, D. Wang, Y. Li, Angew. Chem. Int. Ed. 2017, 56, 6937-6941.
2. D. Malko, A. Kucernak, T. Lopes, *Nat. Commun*. **2016**, 7, 13285.
3. G. Kresse, J. Furthmüller, *Comput. Mater. Sci*. **1996**, 6 , 15-50.
4. M. Bajdich, M. García-Mota, A. Vojvodic, J. K. Nørskov, A. T. Bell, J. *Am. Chem. Soc*. **2013**, 135, 13521-13530.
5. J. K. Nørskov, J. Rossmeisl, A. Logadottir, L. Lindqvist, J. R. Kitchin, T. Bligaard, H. Jónsson, J. Phys.l *Chem. B* **2004**, 108 , 17886-17892.
